# Elevation uncertainties in the Mekong Delta quantified using a transferable approach

**DOI:** 10.1038/s41598-026-38315-y

**Published:** 2026-02-04

**Authors:** Katharina Seeger, Philip S. J. Minderhoud

**Affiliations:** 1 Soil Geography and Landscape Group , Wageningen University & Research , NL-6708PB Wageningen, Netherlands; 2 Institute of Geography , University of Cologne , 50923 Cologne, Germany; 3 Department of Civil Environmental and Architectural Engineering , University of Padova , Padova, Italy; 4 Department of Subsurface and Groundwater Systems , Deltares Research Institute , Utrecht, The Netherlands

**Keywords:** Digital elevation model (DEM), Land elevation change, Land subsidence, Relative sea-level rise, Satellite data, Vertical datum, Climate sciences, Environmental sciences, Natural hazards

## Abstract

The elevation of coastal lowlands relative to local sea level is a crucial determinant for their exposure and is key input for coastal hazard and relative sea-level rise impact assessments. For many data-sparse coastal lowlands worldwide, global digital elevation models are often the only source of information. While these provide an adequate spatial (i.e. horizontal) resolution for regional, delta-wide coastal assessments, their vertical errors in the range of several metres impede investigations of (relative) sea-level rise impact where changes occur on millimetre- to centimetre-scale. Assessing the quality of available elevation datasets is required to identify the best performing model(s) to use for generating reliable coastal impact and exposure assessments. While data-intrinsic inaccuracy has been extensively addressed both in dataset documentation and literature, the relevance and proper vertical datum conversion from global geoid and ellipsoid to local sea level is often still omitted in many applied studies from coastal research. Similarly, the impact of the actuality of elevation data (i.e. time since data acquisition) on assessments in coastal lowlands is so far understudied although elevation models may become quickly outdated, especially where coastal lowlands are facing high rates of elevation change resulting from the interplay of vertical land motion, (vertical) sediment accretion and sea-level change. Particularly for flat, low-lying subsiding coastal landscapes like the Mekong Delta, being in parts only a few decimetres elevated above sea level and experiencing land subsidence of up to several centimetres per year, the reliability of elevation data and adequate representation of elevation relative to local sea level as well as the consideration of factors impacting elevation over time is of utmost importance. We present a transferable and ultimately globally applicable approach to quantify and attribute uncertainties in elevation assessment for data-sparse coastal lowlands using global elevation models to sources such as inaccuracy, vertical datum offset and actuality. The approach combines openly available land elevation and sea-level datasets, integrated with non-linear time series and projections of sea-level change and vertical land motion while also pointing out the need of information on sediment accretion/erosion. We showcase this approach by revisiting land elevation in the Vietnamese Mekong Delta (i) by vertically referencing 11 commonly used global elevation models and an updated local elevation model to a common actual, local sea-level datum, and (ii) by conducting a thorough assessment of elevation model performance that not only allows for the quantification of errors and elevation assessment uncertainties but also their attribution to data-intrinsic inaccuracy, vertical datum offset and, tentatively, non-linear impact of elevation change due to vertical land motion (e.g. extraction-induced land subsidence) and sea-level change affecting the actuality of the elevation model. Our approach not only allows to improve the understanding of coastal elevation to further improve relative sea-level rise and flood impact assessments and to substantiate projections of future elevation in the Mekong Delta, but in its design, applying solely open data and commonly used GIS software, facilitates similar assessments of elevation model performance and elevation assessment uncertainties in other (data-sparse) coastal regions in the world.

**Supplementary Information:**

The online version contains supplementary material available at 10.1038/s41598-026-38315-y.

## Introduction

Coastal lowlands face an increasing risk of sea-level rise (SLR) as global, climate-induced SLR is often accelerated by coastal subsidence^1^. Especially densely populated river deltas are prone to suffer from magnitudes of land subsidence that outpace dimensions of absolute sea-level change and together with sediment starvation due to sediment trapping in upstream dams culminate in elevation loss (e.g., refs^1–6^). Consequently, these lowlands are also increasingly exposed to other coastal hazards such as storm surge flooding (e.g., ref^7)^. The elevation of coastal lowlands above sea level is a critical factor in safeguarding these globally important landscapes from temporary and permanent inundation, salinity intrusion and other cascading effects on the environment and society, affecting for example socio-economic productivity (see also ref^8)^. Land elevation data, mostly in form of digital elevation models (DEMs), are necessary for any quantitative coastal hazard impact assessment or projection, and the reliability of these assessments is strongly determined by the quality of the underlying elevation data. Therefore, thorough DEM accuracy assessments are needed to ensure the reliability of flood risk and SLR impact evaluations or at least to quantify ranges of uncertainty, both in terms of area, population and assets at risk (e.g., refs^9–12^).

Information on land elevation is obtained either by direct measurements through topographical levelling surveys and Global Navigation Satellite System (GNSS) measurements, or by exploiting remote sensing data from aircrafts or satellites (optical, radar, laser altimetry) to generate DEMs of the Earth’s surface. DEMs are subdivided into models representing a landscape’s surface (so-called digital surface models (DSMs)), i.e. including vegetation and building heights, and digital terrain models (DTMs) that represent elevation of the bare earth (e.g., refs^13,14)^. While the DEM type depends on the acquisition techniques and processing approaches of a given dataset, it is important to select the correct DEM type fitting the purpose of application^13^.

Local ground measurements of land elevation data provide elevation information of high vertical accuracy, but suffer from coarse spatial resolutions and time-consuming data acquisition. Through remote sensing techniques such as aerial photogrammetry, and more recently, airborne Light Detection and Ranging (LiDAR), the quality of DEMs has been improved over the years to reach horizontal resolutions and vertical accuracies on centimetre- to decimetre-scale (e.g., refs^15–17^). While such high-accuracy, locally-source data is publicly available for several coastal regions, for example in the United States, Australia, New Zealand and Europe, it is unfortunately not available for major parts of the Earth’s coast, amongst other the vast, densely populated coastal lowlands of Asia and Africa as well as many Small Island Developing States. Here, global, freely available, satellite-based DEMs are commonly used (e.g., as highlighted by refs^18,19^), however often studies do not take limitations of the applicability of these DEMs properly into consideration. Although global DEMs often provide an adequate spatial (i.e. horizontal) resolution (i.e. in the range of ~ 10 to 90 m), sufficient for setting up regional, delta-wide flood models or estimating coastal population and land-use asset exposure, their vertical errors in the range of several metres impede investigations of (relative) SLR impact where changes occur on millimetre- to centimetre-scale. Inaccuracies related to the acquisition of elevation data or DEM processing itself (e.g. sensing or interpolation artefacts) have been addressed by post-processing first-order DEMs such as SRTM^20^, ASTER^21,22^, AW3D (ALOS)^23^ and TanDEM-X^24^, through applying void-filling and smoothing (e.g. ACE2^25–27^, Copernicus DEM^28^, filtering elevation data with spatial vegetation, building and/or applying population data in random forest algorithms or neural networks (MERITDEM^29^, FABDEM^30^, Coastal DEM^31,32^ or employing recently-available satellite LiDAR measurements (ICESat-2) (GLL-DTM^33,34^; DeltaDTM^35^. These recent DEMs constitute the newest generation with improved vertical accuracy, specifically targeting applications in coastal lowland contexts, with the FathomDEM^36^, created using advanced postprocessing of the Copernicus DEM, as the youngest sibling.

The majority of those DEMs is provided with vertical reference to ellipsoid (e.g. WGS84) or a global geoid (e.g. EGM96, EGM2008), the latter often considered to reflect sea level, thus providing elevation information at global scale with respect to sea level. However, the accuracy of these global geoids to represent the actual local sea-level height varies a lot and is dependent on the amount and accuracy of input data used when generated. Offsets with true local sea-level height in several regions worldwide can be more than 1 m, a bias which is very often not corrected for in impact assessments using global DEMs. Consequently, in these data-sparse regions, local sea level may differ up to more than a metre from the global geoid (e.g., refs^11,37–39^), thereby introducing systematic errors into SLR impact assessments conducted based on global elevation data whose vertical reference was not properly corrected from geoid/ellipsoid to sea level. Finally, the actuality of elevation data, i.e. referring back to the time of data acquisition, impacts the quality of coastal impact assessments. Especially in rapidly subsiding coastal lowlands such as river deltas, DEMs may become quickly outdated, as these landscapes can experience several centimetres to even decimetres subsidence per year^6,40–42^. In such areas, using for example the SRTM DEM, originally acquired in February 2000, or any of its post-processed versions in a heavily subsiding environment results in substantial misjudgements of actual elevation, and this limitation is hardly considered in the majority of coastal impact assessments based on global DEMs.

While several studies have addressed DEM accuracy by comparing different DEMs and investigate their performance in terms of fluvial flood inundation (e.g., refs^43–47^), only a few focused on the impact of DEM selection on (relative) SLR impact^11,12,34,38,48^. So far, only Minderhoud et al.^38^ tentatively attributed the errors associated with a DEM in the Mekong Delta to DEM accuracy and the lack of datum conversion to local sea level. However, due to information paucity on local vertical datums, this was done through a basic transformation, i.e. by lowering the global MERITDEM to equalise the mean elevation of a local DEM (TopoDEM) with sea level datum alignment, rather than systematically converting the vertical reference system from the EGM96 geoid to local sea level as indicated by tide gauge data (i.e. Hon Dau tide gauge). Although tide gauges provide highly local information on sea level, their usage is only recommended if they provide a sufficiently long, continuous and up-to-date monitoring period for small study sites or regions without any significant sea-level variations along the coast^49^. Also the absence of any documentation about the position of a tide gauge with respect to a certain geoid/ellipsoid may hamper the proper alignment of a geoid- or ellipsoid-referenced DEM to local sea level. In their DEM accuracy and (relative) SLR impact assessment for the Ayeyarwady Delta (Myanmar), Seeger et al.^11 ^employed an open-data vertical datum conversion approach that – in the absence of any suitable tide gauge information – allows for the proper conversion of global DEMs to actual, continuous local mean sea level (MSL) along the Myanmar coast by applying latest, freely available satellite-altimetry-derived mean dynamic topography (MDT). This workflow also includes the beforehand required correction of geoid offset between the different vertical reference systems used by elevation and sea-level datasets and thus improves previous datum conversion workflows that lack this relevant processing step (e.g., refs^33,34^).

**Fig. 1:**
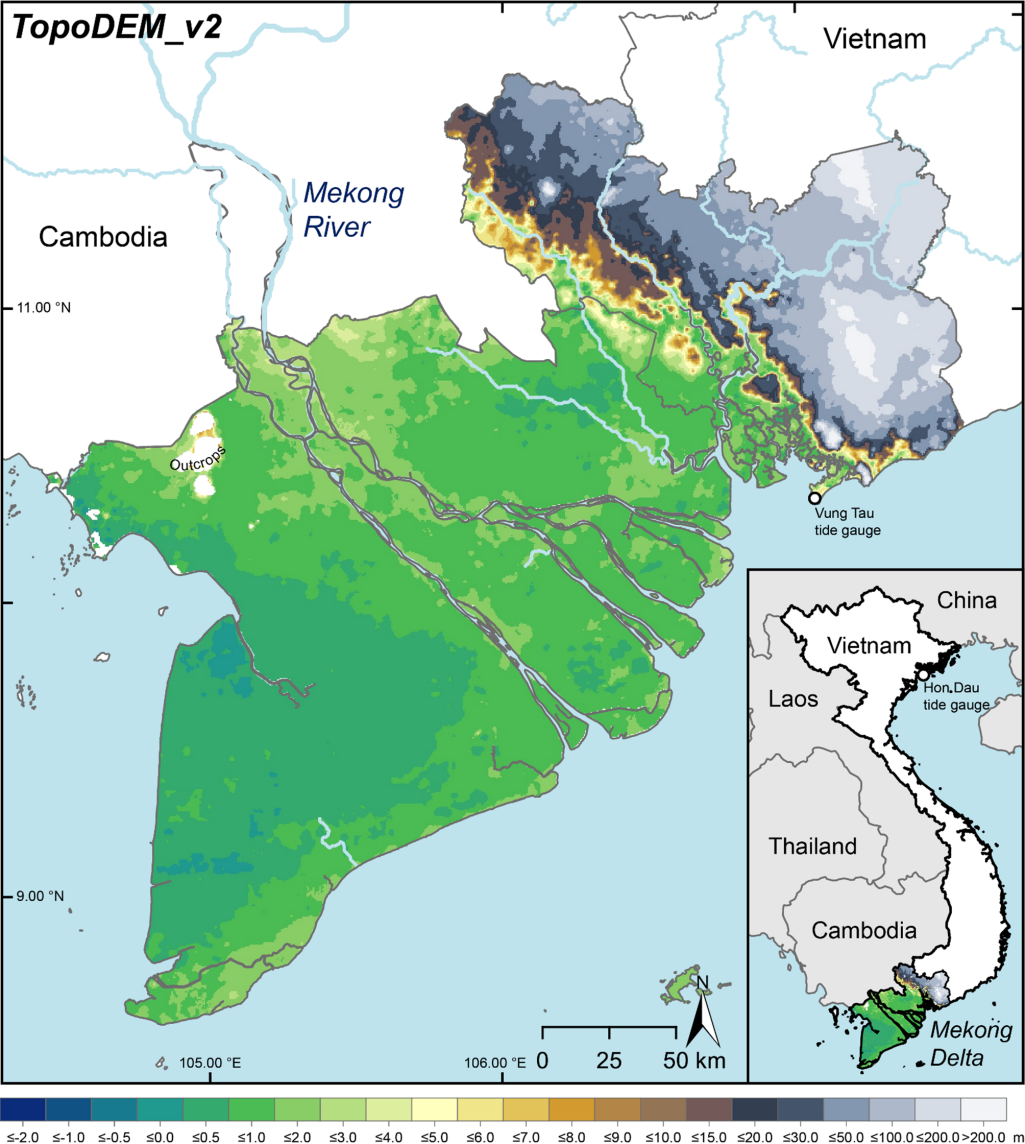
Local elevation model of the Vietnamese Mekong Delta, updated from Minderhoud et al.^38^ by referencing to actual local, continuous mean sea level indicated by mean dynamic topography^51^.

In this study, we investigate land elevation datasets and their performance in data-sparse coastal lowlands, showing a transferable and ultimately globally applicable approach to quantify and attribute uncertainties in global DEMs to sources such as inaccuracy, vertical datum offset and actuality, which matters in terms of relative SLR impact. We highlight the applicability of this approach by revisiting land elevation in the Vietnamese Mekong Delta where Minderhoud et al.^38^ uncovered the delta’s true elevation to be on average only ~ 0.8 m above local sea level whereas previous assessments using global DEMs (e.g. SRTM and MERITDEM) assumed the average elevation of the delta to be considerably higher (i.e. 2.6 m and 3.3 m above sea level, respectively). The previous overestimations stemmed from a combined effect of vertical datum offset, DEM data inaccuracy, and DEM actuality, however the individual contribution of each uncertainty was not quantified^38^, which we will advance upon in this study. We updated the local Topo DEM of Minderhoud et al.^50^ (hereafter referred to as Topo DEM v1), including Ho Chi Minh City and neighbouring provinces, to actual, continuous local MSL by applying an updated vertical datum conversion from Seeger et al.^11^ (hereafter referred to as TopoDEM_v2; Fig. 1). Consequently, we extend the DEM accuracy assessment to evaluate the performance of in total 11 high-resolution, global DEMs, thereby ensuring to include latest available coastal DEMs such as DeltaDTM while also reassessing those DEMs that Minderhoud et al.^38^ investigated in their study (i.e. SRTM and MERITDEM). Furthermore, we investigate the potential impacts of absent and incomplete, incorrect datum conversion, e.g. by including the GLL-DTM v2 referenced to (outdated) MDT as provided by Vernimmen and Hooijer^34^, using the approach of this study. We also consider different versions of recent DEMs and integrate both DeltaDTM v1 and v1.1 as we assume the inclusion of elevations up to 30 m in v1.1 to result in (slight) differences in DEM performance in the flat, low-lying Mekong Delta (see also Pronk et al.^35^). To enable 1:1 comparison to findings from Minderhoud et al.^38^, we focus on results obtained for the Vietnamese Mekong Delta in the main text while providing also the statistics for the extended area of interest in the supplementary material (e.g. Supplementary Figs. 1–3; Supplementary Tables 3–5). Based on this thorough assessment, integrating the DEMs in their original vertical reference and converted to the same vertical datum, our study not only allows to extend and detail previous studies on the Mekong Delta’s elevation but also to attribute and quantify the sources causing uncertainties in the global DEMs to adequately represent the delta’s local elevation. Therewith, we showcase the considerations to be made when handling elevation models in data-sparse local to regional contexts and derive the best compromise of high accuracy and resolution data recommended for further studies on the Mekong Delta such as sea-level rise impact assessments and flood modelling research.

## Results and interpretation

### Converting vertical datum to actual continuous local sea level

The reliability of elevation data in terms of accuracy (i.e. the absence of uncertainties arising from data acquisition and DEM interpolation) and adequate representation with respect to actual continuous local sea level (i.e. the appropriateness of the vertical reference system to equal actual true sea level experienced along a given coastline) is of utmost importance for flat, low-lying coastal landscapes like the Mekong Delta. Being in parts only a few decimetres elevated above sea level makes elevation-dependent assessments highly sensitive to any uncertainties in the elevation data itself and in the estimation of coastal sea level.

**Fig. 2:**
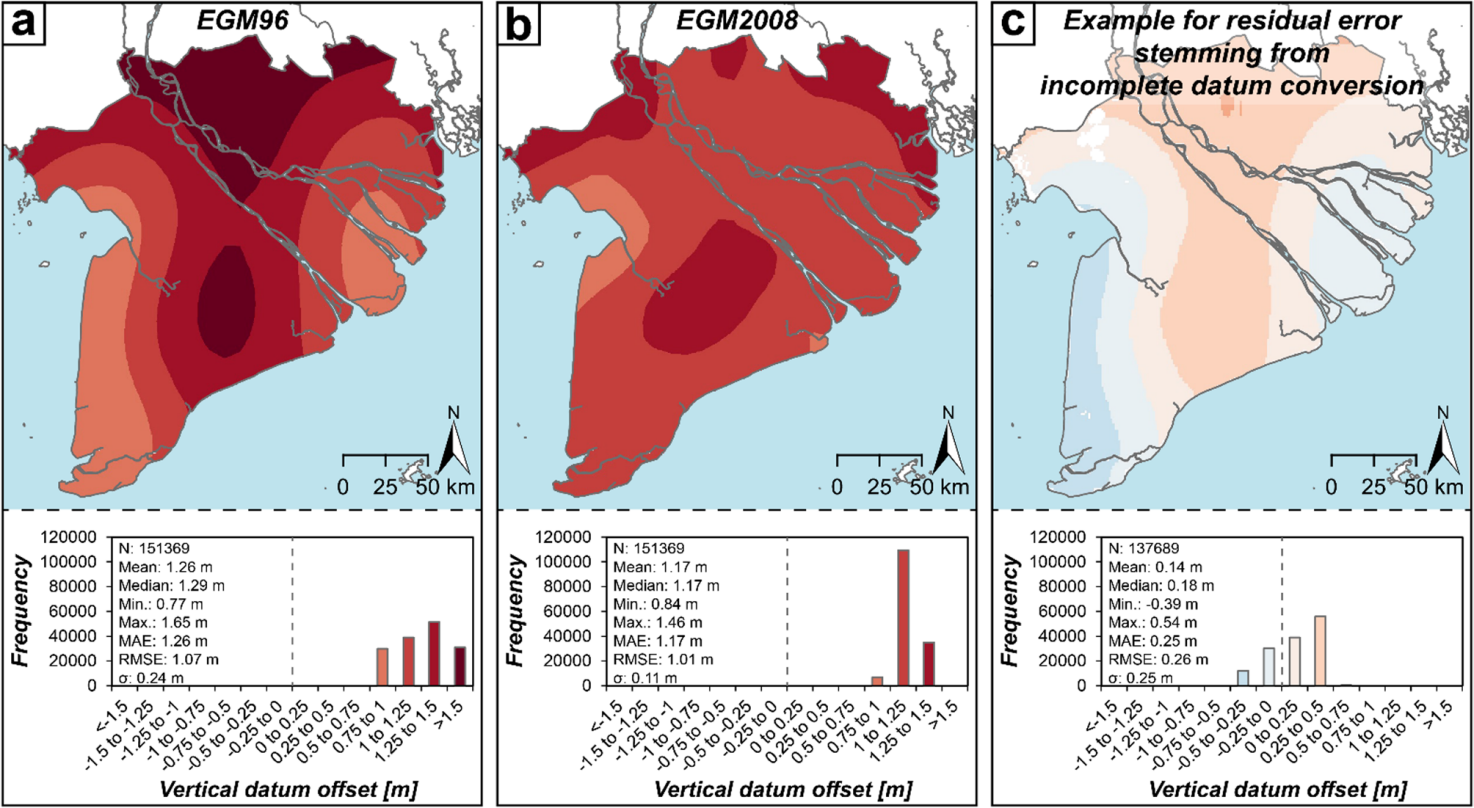
Offsets between commonly used geoid models (EGM96 (**a**); EGM2008 (**b**)) as well as an example of incomplete datum conversion (**c**) and local mean sea level as indicated by mean dynamic topography (MDT) of Jousset et al.^51^ in the Vietnamese Mekong Delta. Sea-level heights above EGM96 and EGM2008 were obtained by applying a conversion of MDT_GOCO06s_ – (EGM96 – GOCO06s) and MDT_GOCO06s_ – (EGM2008 – GOCO06s). The residual error arising from incomplete datum conversion was calculated for the example of GLL-DTM v2^34^ by subtracting the GLL-DTM v2 corrected to MDT (this study) from GLL-DTM v2 referenced to its original vertical datum^34^.

To assess the uncertainties related to vertical reference frames and local sea level for the different assessments and DEMs in the Mekong Delta, we focused on the most common vertical datums used for global DEMs (i.e. EGM96 and EGM2008), which also constitute the original vertical reference of the DEMs used in previous Mekong Delta assessments. We quantified the differences between EGM96, EGM2008 and local MSL, respectively, and determined a mean geoid offset of 1.26 m (median: 1.29 m) for EGM96 and 1.17 m (median: 1.17 m) for EGM2008. Similarly, the offset range is lower for EGM2008 and constitutes only 0.84 m to 1.46 m, with the EGM2008 showing less variability (σ = 0.11 m) while it ranges from 0.77 m to 1.65 m for EGM96 and showing larger deviation of 0.24 m (Fig. 2). This better performance of EGM2008 is probably related to the increased amount of gravitational field data serving as an input and allowing for an improved performance of geoid interpolation in the larger data-sparse region.

We quantify to what extent the vertical datum offset impacts the reliability of an individual global DEM in the Mekong Delta and compare the respective DEMs both in their original reference and transposed to actual continuous local MSL with the local TopoDEM_v2 as well as its local point elevations. We observe that the respective global DEM errors reduce after converting them to actual continuous local MSL (Table 1). Determining the difference between DEM errors before and after datum conversion allows to quantify the impact the vertical datum issue has on the individual DEM reliability. Tendentially, the smaller the overall DEM errors, the larger the impact of lacking vertical datum conversion is. In a spatially resolved comparison with TopoDEM_v2, the discrepancies introduced range from 11.6% to 147.4%. Thereby, percentages of more than 100.0% are obtained in case the uncertainty of the transposed DEM is smaller than the vertical datum offset of the respective original vertical reference system used (Fig. 2) and the difference in DEM error before and after datum conversion exceeds the uncertainty of the transposed DEM (Table 1), specifically for the versions of DeltaDTM (i.e. 101.7% for v1 and 147.4% for v1.1). Similarly, the impact of vertical datum conversion is also high for other DEMs such as CoastalDEM v2.1 (46.3%), FABDEM (48.6%) and MERITDEM (59.7%), thereby indirectly reflecting the effectiveness of accuracy improvement through post-processing which leads to a relative dominance of the vertical datum issue in the DEM-related uncertainty. Similar patterns, however with slightly different percentages, are documented for the comparison with local point elevations, enabling pointwise DEM uncertainty attribution to vertical datum offset over the entire point elevation range as well as by focusing on particularly low-lying elevations (i.e. ≤10 m above MSL).

Besides the absence of vertical datum conversion from a global geoid or ellipsoid model to actual continuous local sea level, another error in handling coastal elevation and sea-level is partial or incomplete, and therefore incorrect, datum conversion. From the global DEMs used in this study, this error was only encountered with the GLL-DTM for which geoid offset correction between the elevation and sea-level data used was not conducted during the processing of the original GLL-DTM^33,34^, thereby resulting in artefacts impacting the alignment and consequently applicability of this DEM. Comparing the original GLL-DTM v2^34^ with the GLL-DTM v2 properly converted to actual continuous local MSL reveals only minor average offsets at large regional to global scale, as smaller-scale offsets in both directions average each other out. However, at local to smaller-regional scale the variability is large and the impact of the incorrect conversion becomes much more relevant (this study). In the Mekong Delta, the incorrect vertical datum conversion affecting the GLL-DTM v2 resulted in offsets of ca. 0.14 m (mean) and 0.18 m (median), with a standard deviation of 0.25 m and overall ranging from −0.39 m to 0.54 m (Fig. 2C). RMSE of GLL-DTM v2 improves by ca. 0.12 m (33.3%) (0.08 m (17.6%) in comparison with local spot heights) if datum conversion is conducted completely and correctly. Consequently, errors made in vertical datum conversion account for 33.3% (17.6% in comparison with local spot height) of the DEM’s uncertainty (Table 1). With a higher spatial resolution of GLL-DTM, however, we would expect substantially greater discrepancies and effects on the quality of the DEM attributable to improper handling of the elevation and sea-level data.

### Performance of digital elevation models in the Vietnamese Mekong Delta

#### Revisiting local elevation data in the Vietnamese Mekong Delta

**Fig. 3:**
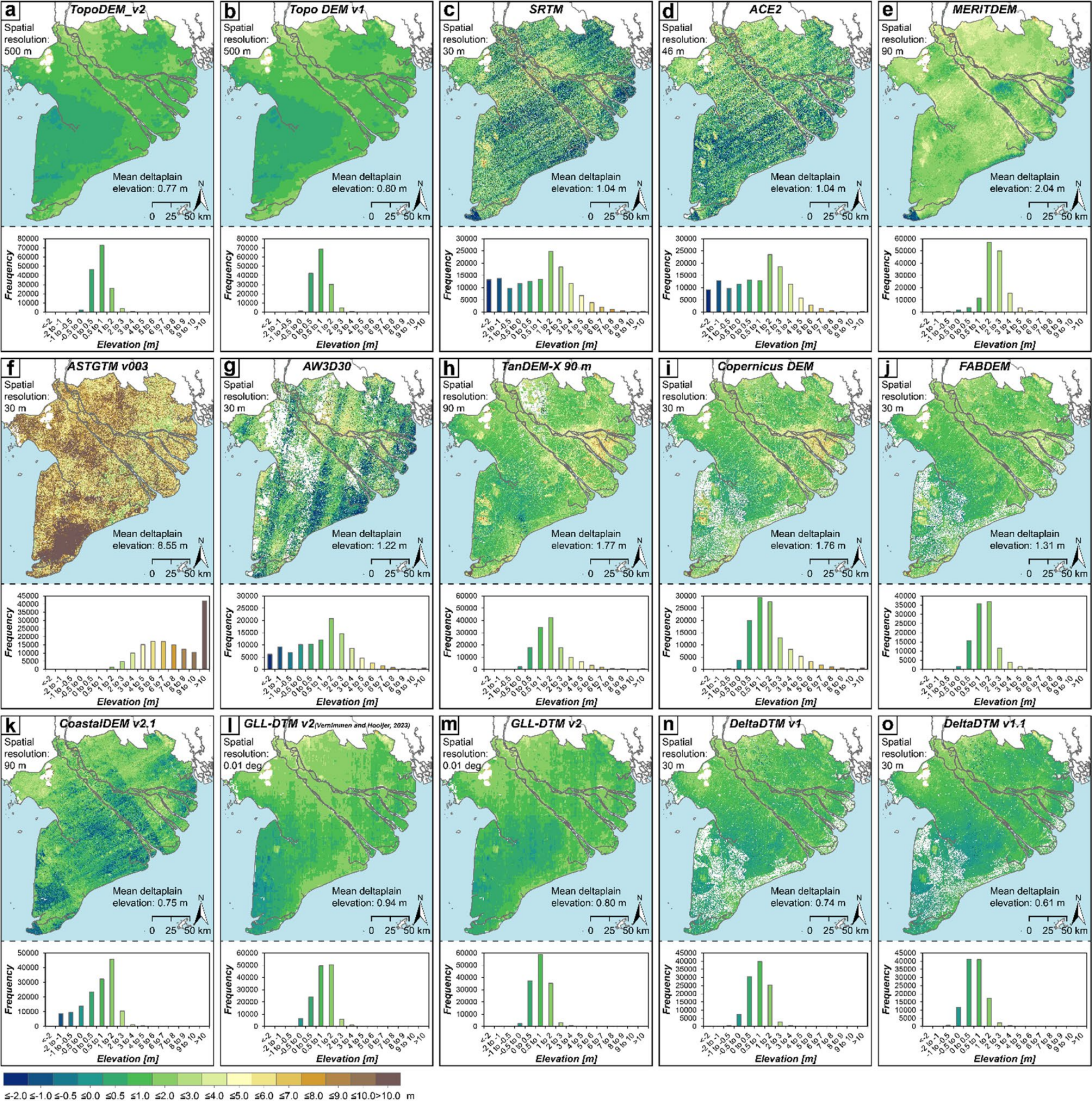
Digital elevation models included in this study, all referenced to actual, local continuous mean sea level as indicated by mean dynamic topography^51^.

**Fig. 4:**
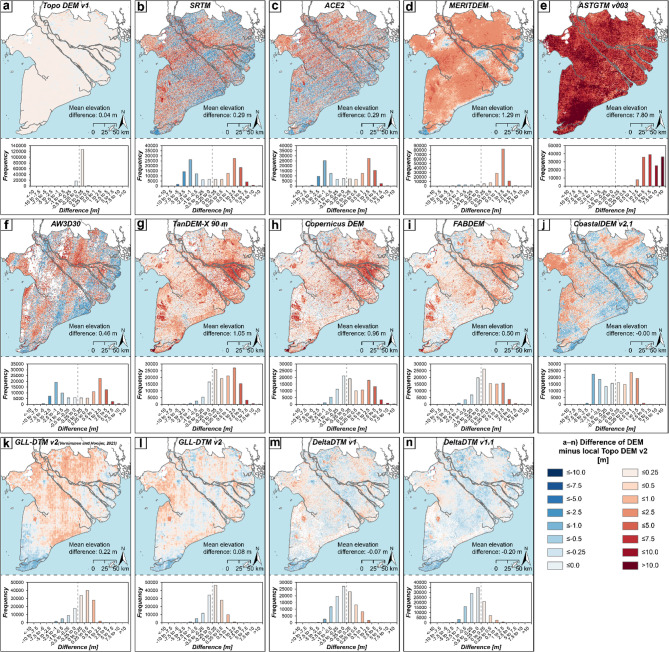
Height residuals of DEMs referenced to mean sea level compared to TopoDEM_v2.

We updated the existing Topo DEM v1^50^ by referencing the data to actual, continuous local MSL (TopoDEM_v2) which enables for an up-to-date assessment of land elevation in the Vietnamese Mekong Delta. Based on visual impression, the overall reflection of land surface height changes only slightly (Figs. 1 and 3). The frequency of lower elevations and thus the spatial extent of lowly elevated areas increased. This becomes particularly evident in the Ca Mau peninsula, reflecting more pronounced sea-level variations in this part of the sea (especially in Rach Gia Bay) compared to Hon Dau in northern Vietnam, resulting in higher MSL/lower elevation above MSL. While in comparison the maximum and minimum elevations increased and decreased for TopoDEM_v2, the average elevation of the Mekong Delta is updated to 0.77 m (mean) and 0.72 m (median) compared to 0.80 m (mean) and 0.74 m (median) as previously indicated by Topo DEM v1, resulting in an average height residual of 4–5 cm (Fig. 4). The fact that this amount equals annual sinking rates of land subsidence hotspots in the Mekong Delta^40,41^ highlights the enormous sensitivity of the flat, low-lying and sinking landscape to any error and uncertainty in the elevation data, even if high-accuracy local elevation data is used. Consequently, if properly conducted, converting the vertical datum from MSL indicated by a single tide gauge (and established several years ago) to actual, continuous sea-surface height along the deltaic coast makes the characterisation of local elevation with respect to sea level more precise. This improvement is due to the possibility to account for potential sea-level variations along the ca. 2000 km long Vietnamese coastline. In the Mekong Delta, this is a crucial step to further narrow down existing uncertainties in the assessment of relative SLR. Topo DEM v1 and TopoDEM_v2 have been validated by local point elevations and are accurate to 0.26 m RMSE (Supplementary Tables 1, 2, 4 and 5).

#### Which one is the best? – On the local validation of global satellite-based digital elevation models

Disentangling DEM uncertainty into DEM-specific accuracy and vertical referencing to actual (continuous) local sea level is crucial to understand the suitability and therefore reliability and applicability of a DEM in a given coastal study area for any coastal hazard or impact assessment. With all elevation data (both DEMs and local point elevations) in the same vertical reference frame, i.e. MSL according to MDT^51^, the impact of respective vertical datum offset on the DEMs’ quality assessment is excluded. Hence, we can characterise and compare their performance and accuracy against the context of their generation (i.e. including data acquisition, DEM interpolation and further processing).

Visual inspection of the DEMs available for the Mekong Delta reveals the huge differences in their capability to correctly reflect the delta’s flat, low-lying terrain (Fig. 3). Rather, artefacts like stripes from sensing are dominating, especially in SRTM, ASTGTM v003 and AW3D30, and are also preserved in post-processed DEMs such as ACE2, MERITDEM and CoastalDEM v2.1, reflecting that the application of smoothing filters and correction algorithms did not perform well in this coastal lowland (Fig. 3). Consequently, the elevation frequency distributions of those DEMs show an almost bimodal shape and are characterised by one mode in the range of 1 m to 2 m (i.e. reflecting the delta’s average elevation) and a second, minor one at the lower end of the frequency distribution with most values in classes − 1 m to −2 m and <−2 m (i.e. including erroneous values resulting from the DEM’s artefacts) (Fig. 3). Stripes related to the SRTM DEM are largely hidden in MERITDEM and likely do not become visible in its elevation frequency distribution as related values are in the same range as the indicated delta elevation. While average elevations of SRTM and MERITDEM prior to vertical datum conversion are corresponding with values determined by Minderhoud et al.^38^ (for SRTM only, mean delta elevation is ~ 0.3 m lower, likely resulting from slightly different pre-processing performance), they are 0.45 m and 1.06 m lower after transposing them to MSL, respectively (Fig. 3; Table 1). ASTGTM v003 varies from SRTM-based DEMs and AW3D30 as it indicates the highest elevations throughout the entire array of datasets. In contrast, visual comparison of TanDEM-X 90 m and its post-processed versions, such as Copernicus DEM and FABDEM, reveal a similar representation of the delta terrain, which is also in line with the local DEM, but indicate higher mean elevations. Developed for improving global DEM performance in coastal lowlands, the global coastal DEMs of CoastalDEM v2.1, GLL-DTM v2, and DeltaDTM v1 and v1.1 show an overall better representation of the Mekong Delta’s elevation, both in terms of approaching average elevation and elevation frequency distribution as well as in lower error statistics (Fig. 3; Table 1). All of these coastal DEMs have been trained on or processed with high-accuracy ICESat-2 satellite LiDAR. However, CoastalDEM v2.1 still suffers from underlying SRTM artefacts (which are transferred via NASADEM that was used as source DEM for generating CoastalDEM v2.1) although it reflects the delta’s average elevation well. Consequently, indicated elevations are characterised by a left-skewed frequency distribution and comparably high standard deviation which is twice as high as for other coastal DEMs (Table 1). Both GLL-DTM v2 and DeltaDTM yield a more accurate representation of the delta’s terrain. Especially GLL-DTM v2 (converted to MSL by this study) and DeltaDTM v1 reveal the best results, with mean errors less than 0.10 m and RMSE of 0.35 m and 0.49 m, respectively (Table 1).

**Table 1:** Performance of local and global DEMs in the Vietnamese Mekong Delta validated by the local TopoDEM_v2 and DEM-specific vertical datum offsets quantified as differences in RMSE and discrepancies from RMSE of DEMs referenced to mean dynamic topography (MDT). The statistics were extracted from DEMs masked for water bodies and outcrops and resampled to 500 m × 500 m spatial resolution. Discrepancy was calculated as RMSE of DEM_MDT_ minus RMSE of DEM_original datum_, divided by RMSE of DEM_MDT_ and expressed in %. N – number of grid cells in the study area, with no-data values excluded for each DEM, respectively; Mean DEM – mean DEM elevation in the study area; Median DEM – median DEM elevation in the study area; Min. DEM – minimum DEM elevation in the study area; Max. DEM – maximum DEM elevation in the study area; σ DEM – standard deviation of DEM elevation in the study area; HR – height residual; MAE – mean absolute error; RMSE – root mean square error.

DEM	*N*	Mean DEM (m)	Median DEM (m)	Min. DEM (m)	Max. DEM (m)	σ DEM (m)	Max. negative HR (m)	Max. positive HR (m)	Mean error (m)	MAE (m)	Median error (m)	RMSE (m)	Difference in RMSE (m)	Discrepancy (%)
TopoDEM_v2	152,028	0.77	0.72	−0.37	8.97	0.57								
Topo DEM v1	147,582	0.80	0.74	−0.52	6.98	0.54	−7.03	1.62	0.04	0.06	0.05	0.10		
SRTM_MDT_	144,113	1.04	0.91	−6.89	143.06	2.56	−10.12	142.12	0.29	1.87	0.17	2.55	0.45	17.6
SRTM_EGM96_	145,672	2.27	2.00	−7.00	145.00	2.63	−9.56	144.06	1.52	2.26	1.40	3.00		
ACE2_MDT_	133,927	1.04	0.93	−6.99	140.23	2.32	−8.90	139.29	0.29	1.65	0.17	2.26	0.50	22.0
ACE2_EGM96_	134,211	2.30	2.20	−7.00	141.70	2.38	−10.02	140.76	1.55	2.09	1.55	2.76		
MERITDEM_MDT_	146,263	2.04	1.96	−6.65	139.47	1.33	−7.89	138.53	1.29	1.40	1.28	1.80	1.06	58.7
MERITDEM_EGM96_	146,269	3.29	3.26	−6.46	140.94	1.40	−7.44	140.00	2.54	2.57	2.56	2.86		
ASTGTM v003_MDT_	145,981	8.55	7.44	−1.43	152.33	4.99	−3.88	151.39	7.80	7.80	6.68	9.30	1.08	11.6
ASTGTM v003_EGM96_	145,981	9.80	9.00	0.00	154.00	5.00	−2.60	153.06	9.05	9.05	7.98	10.38		
AW3D30_MDT_	109,349	1.22	1.00	−6.96	111.90	2.46	−8.79	111.36	0.46	1.72	0.29	2.46	0.54	22.1
AW3D30_EGM96_	110,184	2.46	2.00	−7.00	113.00	2.54	−9.64	112.47	1.70	2.22	1.52	3.00		
TanDEM-X_MDT_	138,263	1.77	1.24	−6.81	163.51	1.85	−7.81	162.57	1.05	1.15	0.48	2.05	0.83	40.6
TanDEM-X_EGM96_	138,313	3.02	2.52	−6.51	164.98	1.86	−7.66	164.04	2.29	2.30	1.75	2.88		
Copernicus DEM_MDT_	114,885	1.76	1.09	−5.35	138.51	2.03	−6.40	137.57	0.96	1.18	0.24	2.19	0.71	32.4
Copernicus DEM_EGM2008_	114,887	2.94	2.29	−4.27	139.93	2.02	−5.23	138.99	2.14	2.15	1.42	2.90		
FABDEM_MDT_	108,892	1.31	1.01	−4.09	139.50	1.41	−5.00	138.55	0.50	0.66	0.21	1.43	0.69	48.6
FABDEM_EGM2008_	113,820	2.48	2.21	−2.55	144.54	1.38	−3.50	143.60	1.68	1.69	1.40	2.12		
CoastalDEM v2.1_MDT_	145,917	0.75	0.77	−5.50	159.20	1.26	−6.80	158.26	−0.00	0.77	0.03	1.20	0.56	46.3
CoastalDEM v2.1_EGM96_	146,009	2.01	2.01	−4.05	159.96	1.32	−5.35	159.02	1.26	1.38	1.32	1.76		
GLL-DTM v2_MDT (this study)_	137,689	0.80	0.76	−1.48	8.09	0.51	−5.41	6.86	0.08	0.26	0.08	0.35	0.12	33.3
GLL-DTM v2_MDT (original)_	138,749	0.94	0.89	−1.67	8.15	0.64	−5.37	6.93	0.22	0.37	0.26	0.47		
DeltaDTM v1_MDT_	107,553	0.74	0.67	−3.21	7.87	0.63	−5.13	6.56	−0.07	0.35	−0.07	0.49	0.72	147.4
DeltaDTM v1_EGM2008_	114,856	1.91	1.83	−1.88	9.56	0.63	−3.98	7.98	1.11	1.13	1.11	1.21		
DeltaDTM v1.1_MDT_	114,585	0.61	0.53	−6.08	28.70	0.69	−7.05	27.64	−0.20	0.33	−0.19	0.55	0.56	101.7
DeltaDTM v1.1_EGM2008_	114,587	1.78	1.69	−4.88	30.00	0.70	−5.84	29.06	0.98	1.00	0.98	1.11		

By subtracting TopoDEM_v2 from each of the other DEMs referenced to MSL, we quantify all differences between local and global elevation datasets. This substantiates the inappropriateness of SRTM, ACE2 and AW3D30 to be applied in the Mekong Delta as inaccuracies are in the range of several metres and are particularly evident from −2.5 m to −1 m and from 1 m to 2.5 m (Fig. 4). Given their bimodal character, these inaccuracies do not show up in any spatial-averaged height residual statistics but only standard deviations and RMSE in the range of ≥ 2 m (Fig. 4; Table 1). Aside from ASTGTM v003, whose elevation is in many parts of the Mekong Delta more than 2.5 m higher than indicated by TopoDEM_v2 (with inaccuracies of more than 10 m, particularly in the southwestern Ca Mau peninsula), also MERITDEM represents the Mekong Delta on average 1.29 m higher (Fig. 4; Table 1). This is surprising as MERITDEM was created based on SRTM and involved a correction for vegetation heights^29^. Thus, we would have expected similar or lower offsets to TopoDEM_v2. However, it rather seems that vegetation was not effectively eliminated while artefact correction and smoothing was applied. Also the TanDEM-X based elevation models reveal higher elevations than TopoDEM_v2. Seeing the sequence of DEM generations from TanDEM-X 90 m to Copernicus DEM to FABDEM, involving corrections such as void filling and vegetation removals, their comparison with local elevation data reveals their improvement in accuracy, particularly in terms of lowered mean errors and average height residuals and especially following vegetation correction in FABDEM (Fig. 4; Table 1). While difference mapping for GLL-DTM v2 (Fig. 4k and l) shows how inadequate consideration of geoid offset in vertical datum conversion also affected coastal inland elevation, DeltaDTM as the most recent coastal DEM, generated by integrating ICESat-2 satellite LiDAR into Copernicus DEM, reveals the overall best performance. DeltaDTM benefits from some of the lowest inaccuracies, small height residuals and low standard deviations while offering a high resolution of 30 m × 30 m. To further investigate the performance of DeltaDTM, and see whether the inclusion of elevations up to 30 m in v1.1 has an impact on its performance in the low-lying Mekong Delta, we included both available v1 and v1.1. We find that although both versions perform very similar, they show slight differences as DeltaDTM v1 outperforms v1.1 in overall lower height residuals and slightly higher accuracy (RMSE = 0.49 m (v1), RMSE = 0.55 m (v1.1). However, since DeltaDTM v1.1 included higher elevations, we would expect its height residuals to be similar to or higher than those for v1, i.e. indicating elevations also similar to or higher than TopoDEM_v2. Instead, DeltaDTM v1.1 is on average 0.20 m lower than TopoDEM v2 and 0.13 m lower than DeltaDTM v1. These are considerably larger differences than the minor, global differences in the range of ~ 2 cm based on the global-scale validation comparison of Pronk et al.^52^.

### Attributing uncertainties in elevation assessment

**Fig. 5:**
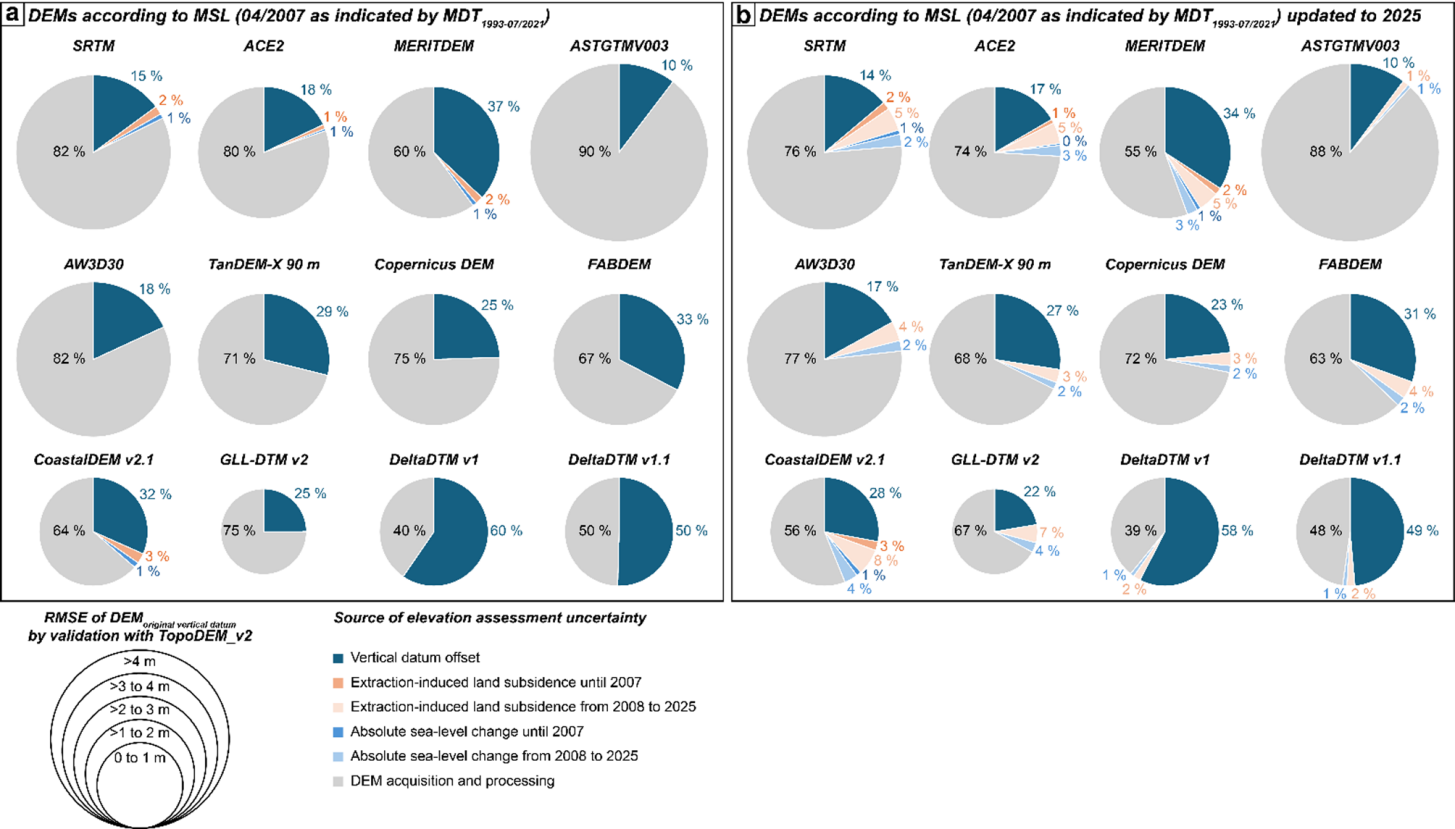
Quantification and attribution of uncertainties associated with global DEMs in their performance to correctly quantify the elevation of the Vietnamese Mekong Delta. Quantifications were conducted relative to root mean square error (RMSE) of DEMs in comparison to TopoDEM_v2 based on their original vertical reference. Sources of elevation assessment uncertainty include vertical datum offset, inaccuracy resulting from DEM acquisition and processing, as well as time since DEM generation over which land subsidence and sea-level change have resulted in elevation change. (**a**) Relative uncertainties for DEMs referenced to mean sea level as indicated by mean dynamic topography (MDT), which provide the local sea level average over the period 1993–07/2021, corresponding to April 2007. (**b**) Relative uncertainties for DEMs referenced to mean sea level as indicated by mean dynamic topography (MDT), including land subsidence and sea-level rise since April 2007. For these tentative calculations, simulated, non-linear, extraction-induced land subsidence^56,57^, tide-gauge observations^58^ and IPCC-projections of sea-level rise^59–61^ were used.

Land elevation is not static but dynamic over time (e.g. following vertical land motion and/or sediment accretion) and the vast majority of coastal lowlands such as river deltas and coastal plains are prone to land subsidence, which may reach several centimetres to locally even decimetres per year (e.g., refs^6,53–55^), together with global SLR resulting in elevated relative SLR^4^. Consequently, the capability of a DEM to adequately represent coastal-deltaic land elevation will change significantly over years as accumulated subsidence together with SLR progress and potentially even exceed the DEM-inherent uncertainty. Therefore, the actuality of the elevation data used is as crucial as the actuality of sea-level information in order to guarantee up-to-date assessments of coastal hazards and impacts. However, this factor is often overlooked as still many assessments apply elevation data which was originally collected ten to even 25 years ago (e.g., as highlighted in Hawker et al.^45^). We here attribute and quantify the sources causing uncertainties in global DEMs to adequately represent the Mekong Delta’s local elevation (Fig. 5). We address the impact of data-inherent inaccuracy, vertical datum offset and data actuality as a consequence of relative sea-level change over time. We include both land subsidence and SLR, by using simulated, non-linear extraction-induced land subsidence^56,57^ (B1 scenario of Minderhoud et al.^57^) and annual rates of local, delta-average SLR estimated from PSMSL data of Vung Tau tide gauge^58^ and IPCC AR6 total rates of sea-level change^59–61^ (Supplementary Table 6).

Unravelling and quantifying the specific contributions of the different factors to uncertainties of elevation assessments using global DEMs, has, to our knowledge, not been performed before. Such an assessment not only reveals which DEMs perform best for a certain area but also pinpoints errors in DEM processing steps (e.g. omitting vertical datum conversion). It also indicates how much DEM performance (and its applicability) can be improved if certain processing steps are prioritised and properly applied. Furthermore, the integration of spatio-temporal data allows for detailing quantifications in elevation assessment uncertainties with elevation change in space and time. With MSL as indicated by MDT^51^ (i.e. as of 2007), for example the RMSE of ACE2 and MERITDEM are very similar if used in the EGM96 geoid as they are provided^27,29^. However, our assessment highlights that vertical datum conversion to MSL in the Mekong Delta is much more effective to improve the results when using the MERITDEM, while for ACE2 still 80% of the documented uncertainties is controlled by inaccuracy in the DEM generation process (i.e. related to acquisition and DEM interpolation) (Fig. 5a). This means that even after proper referencing to sea level, land elevation will be substantially overestimated by ACE2 by several metres (Fig. 5a; Table 1). For global DEMs that included accuracy improvements like vegetation removal or integration of terrain data in their processing (e.g., FABDEM, CoastalDEM v2.1, DeltaDTM v1 and v1.1), vertical datum offset and consequently the need for conversion mount up to 30% to 60% uncertainty. This reflects – at least for DeltaDTM – vertical datum offset and need for conversion as an either equal or the most critical factor to consider in order to improve DEM performance. As this attribution highly depends on the magnitude of datum offset to local sea level, which varies around the world, as well as local spatial patterns and landscape features impacting the accuracy of DEM processing and post-processing, this attribution does not allow to be simply transferred to other areas and requires a site-specific evaluation following our presented approach.

For the local elevation model in the Mekong Delta, the attribution of uncertainties depends on the respective validation. While data-inherent inaccuracies are documented for the validation by local point elevations, these are attributed to the impact of DEM interpolation (Supplementary Fig. 4). In contrast, these inaccuracies can be ruled out for validation by TopoDEM_v2 due to model consistency of Topo DEM v1 and TopoDEM_v2 and consequently reveals vertical datum offset as the main source of elevation assessment uncertainty, in line with best performing global DEMs (Fig. 5a; Supplementary Fig. 4).

**Fig. 6:**
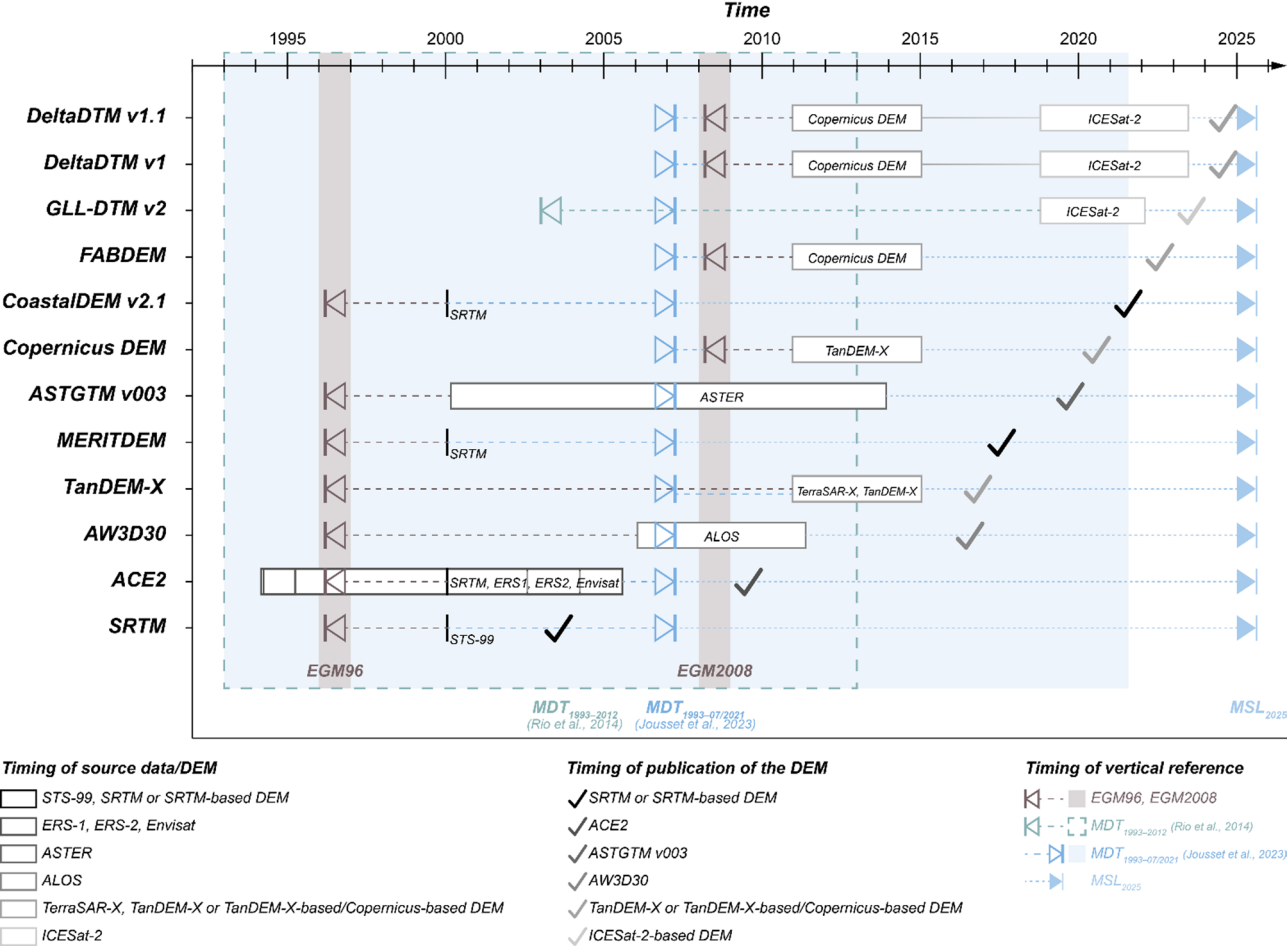
Timeline for data acquisition and publication of global digital elevation models (DEMs) investigated in this study as well as their original vertical reference systems and updated vertical reference to local mean sea level (MSL) as indicated by mean dynamic topography (MDT). Note that although EGM2008 geoid provides a slightly more recent datum than the average of latest available continuous local sea-level information, which MDT^51^ indicates as it dates around April 2007, the geoid does not reflect local sea level well in many data-sparse coastal lowlands. The check icons of this figure were obtained from Font Awesome Free v7.1.0 (CC-BY 4.0 License).

The fact that some of the more recently published DEMs constitute post-processed elevation data such as SRTM, which was acquired in 2000, may cause confusion as users may consider the post-processed DEM to represent more recently acquired elevation data while these DEMs still rely on original, earlier acquired elevation data, leaving the potential impacts of post-acquisition elevation dynamics unaddressed (Fig. 6). We demonstrate the potential impact of outdated elevation on DEM performance using the Mekong Delta and updated the DEMs referenced to continuous local MSL as indicated by MDT (i.e. as of 2007) to MSL 2025. For SRTM and its follow-up versions, consideration of extraction-induced land subsidence and absolute SLR since 2000 until MSL of ~ 2007 already contributes 3–4% to overall elevation assessment uncertainty, while relative SLR until 2025 increases elevation assessment uncertainty relatively by 9–16% (Fig. 5). The impact is less for most recent, TanDEM-X based DEMs (3–6%) until its dimension approaches that of vertical datum offset and accuracy as GLL-DTM v2 shows a smaller vertical datum offset than DEMs originally referenced to a global geoid and reveals a comparably high accuracy (Fig. 5; Table 1). Only for ASTGTM v003, which suffers from the largest inaccuracies, consideration of land elevation and sea-level change does not result in remarkable performance improvement. Compared to the global DEMs, elevation uncertainties for the local TopoDEM v1 and v2 are mainly sourced in the impact of land subsidence and sea-level rise, highlighting the need to adequately account for these processes in order to warrant its outstanding performance in the future.

## Discussion, conclusions and outlook

The previous elevation assessment of the Vietnamese Mekong Delta by Minderhoud et al.^38^ set a benchmark by unravelling the delta’s elevation relative to local sea level and its exposure to relative SLR, which had been grossly underestimated in previous SLR impact assessments based on global DEMs, as they overestimated the delta’s elevation to sea level by several metres. In addition, this work sensitised the coastal research community for proper converting the vertical datum to local sea level by conducting a tentative vertical datum conversion for one of the two assessed global DEMs. In recent years there have been considerable advancements in scientific research on data-sparse coastal lowlands for regions in the world where high-quality data (e.g. LiDAR) is limited or unavailable. The recent advancements range from the publication of newly processed DEMs specifically targeting global coastal lowlands^32–35^, raising awareness on considerations to be made about elevation assessment uncertainty due to DEM inaccuracy and vertical datum offsets (e.g., refs^62,63^) and introducing approaches and showcases how to handle elevation data properly in data-sparse coastal lowlands^9,11,64^. Building on these new DEMs and insights we revisited the land elevation in the Vietnamese Mekong Delta, which provides an excellent test-case being a lowly-elevated coastal landscape with local validation data (i.e. now with TopoDEM_v2) available, thereby updating and extending the previous elevation assessment of the delta^38^.

We took the majority of currently available global DEMs, including the older, most commonly used DEMs and the latest generation of DEMs specifically designed to target coastal lowlands. We compared them against a vertically high-resolution DEM from local origin (i.e. TopoDEM_v2), with all DEMs properly referenced to a common vertical datum, in our case to actual local continuous MSL (as indicated by MDT^51^). In addition, we also compared all DEMs while omitting the necessary vertical datum conversion (i.e. DEMs are kept in their original reference system, which is mostly a global geoid). This is to mimic and evaluate the impacts of the common, erroneous practice present in the majority of global coastal impact assessments (highlighted in Minderhoud et al.^38^). This approach not only allowed us to characterise the presently best performing DEMs but – by the integration of non-linear rates of sea-level change and extraction-induced subsidence – also to attribute and quantify the sources causing uncertainties in a global DEM to adequately represent the delta’s local elevation.

We deem our integration of vertical land motion and sea-level change for the MekongDelta only as a first, preliminary attempt of considering and estimating the effect of elevation dynamics on coastal land elevation datasets. Though we consider the non-linear spatio-temporal behaviour of land subsidence in the Mekong Delta, this assessment only accounts for moderate rates of simulated subsidence induced by groundwater extraction^56,57^ but does not include contributions from other origins such as natural compaction^65,66^, other land-use induced shallow processes^55^, or urban differential compaction^67^. The assessment also does not consider sediment aggradation, but current conditions of sediment starvation suggest the potential to build elevation through sedimentation is very limited^68^. This leaves ample opportunity for future research to consider the spatio-temporal behaviour of the above drivers for better understanding elevation change and to complete the picture of components driving elevation change and controlling related uncertainties in DEMs. Our current assessment provides a first demonstration of the entire picture of uncertainties related to elevation data in coastal lowland contexts and how these uncertainties impact the individual DEMs relatively. To further substantiate these quantifications will require to address the limitations mentioned above and to consider process- and data-driven spatial variability^40,55,57^ (Fig. 4). Observational studies reveal that contemporary, total subsidence rates in the Mekong Delta are in places considerably larger than only previously simulated extraction-induced subsidence and delta-wide averages, up to 5–6 cm/yr^6,40,41,69^. Therefore, we expect relative SLR to have a much higher relative impact on elevation assessment uncertainty, especially in case outdated (global) DEMs are used. While some studies handle elevation in the low-lying Mekong Delta more carefully, for example by using the local Topo DEM of Minderhoud et al.^38^, more recent global DEMs, or addressing vertical datum conversion tentatively (e.g., refs^70–73^), a considerable number of studies still apply outdated and/or inaccurate DEMs that suffer from artefacts (e.g., refs^74–79^), a phenomenon we also observed for coastal hazard and impact assessments in other regions of the world (e.g. refs^80–82^).

Apart from the shortcomings related to the capability to capture the spatio-temporal behaviour of all elevation change components, limitations of our study may lie in the quality of the global-scale, satellite altimetry-based sea-level (MDT) information used as well as technical aspects of the decreasing extrapolation performance of the MDT data with increasing inland distance from the coastline, the applied distance threshold, and the assumptions made in the vertical datum conversion of the local elevation data (see also Methods section). Although global MDT data may provide less accurate locally specific sea-level information than tide gauges, inaccuracies range within a few centimetres (e.g. refs^83,84^), and it allows for continuous sea-level information which is particularly valuable to account for sea-level variations in large coastal landscapes such as deltas and coastal plains. It further serves as a substitute when tide-gauge based sea-level records are not available, too short, incomplete or outdated. To check for the accuracy of the MDT data along the Mekong Delta coast, we compared the satellite-altimetry measurements of sea level that are part of the MDT product with those of Vung Tau tide gauge, which is closest to the delta. The residuals are on average very low and the altimetry observations fit well with the tide gauge record (R^2^ = 0.9), thereby confirming the suitability of the MDT dataset to be applied (Supplementary Fig. 5; Supplementary Table 7). As the use of MDT as a sea-level reference for land elevation data requires its extrapolation over land, other potential limitations like the selection of the extrapolation technique as well as the extrapolation distance, for which the vertical datum conversion approach is deemed valid, may have an impact on the performance on the converted elevation datasets. While we identified the extrapolation approach based on tests of interpolation algorithms, and following previous studies^33,34^, we did not test systematically for the sensitivity of our results towards the distance threshold applied. However, as we deem our approach particularly relevant for coastal lowlands, we consider a distance threshold of 500 km as a reasonable compromise between MDT extrapolation performance while also including the largest coastal lowland in the world, i.e. the Ganges-Brahmaputra-Meghna Delta. Quantifying uncertainties in elevation assessment related to vertical datum offset is comparably straightforward for elevation data referenced to reference frames such as commonly-used global geoids or sea-level datasets as the information needed to perform the required alignment of the respective vertical reference systems in the datum conversion workflow is openly available and well documented (e.g. ref^85^). However, the conversion of datasets referenced to a locally specific sea-level reference (e.g. tide gauge), which may be located far off the coastline under study, to a spatially continuous sea-level reference frame (e.g. MDT) may involve assumptions to be made. For the Mekong Delta, we had to assume that MSL as given by Hon Dau tide gauge (ca. 1400 km north of the delta) equals MSL as indicated by MDT, if updated by accounting for SLR at the tide gauge location since the local datum establishment (i.e. 0.027 m; see Methods section). Our second assumption was that the difference between MSL at Hon Dau tide gauge and GOCO06s geoid at the tide gauge location (= 1.173 m, SD = 0.005 m) is the same difference between geoid (GOCO06s) and local MSL for the Mekong Delta. While there is to our knowledge no possibility to check the validity of our first assumption, we can confirm our second assumption based on the offset between MSL at Vung Tau tide gauge and GOCO06s geoid (= 1.163 m, SD = 0.009 m) which is in the same range as for Hon Dau. Consequently, we deem offset calculations for tide gauges/benchmark locations closer to the study area as valuable cross-check to justify the application of an offset value to transpose datasets from a locally specific sea-level reference, which is located outside the study area (as might be the case when national datums are used), to a spatially continuous reference frame. This applies in particular to countries/regions that use a single (national) datum for extensive coastlines along which sea level and/or geoid variabilities may occur.

With our revisit of land elevation in the Mekong Delta we provide an exemplar showcase of a globally applicable approach to attribute uncertainties in elevation assessment for data-sparse coastal lowlands using global elevation models. Our assessment not only details previous studies on the delta’s elevation further, moreover it highlights how to handle coastal elevation and sea-level data properly: (i) by vertically referencing the DEMs to a common actual, local sea-level datum, and (ii) by conducting a thorough assessment of DEM performance that not only allows for the quantification of errors and elevation assessment uncertainties but also their attribution to DEM inaccuracy, vertical datum offset and, tentatively, non-linear impact of elevation change due to vertical land motion (e.g. extraction-induced land subsidence) and sea-level change affecting DEM actuality. This improved understanding of coastal elevation serves as a starting point to further improve impact assessments of flooding and relative SLR (e.g., refs^38,73^) and substantiating projections of future elevation in the Mekong Delta^57^. With all quantifications of DEM performance and vertical datum offsets based solely on open data and approaches that can be applied in any GIS environment, our demonstrated approach may set a benchmark to initiate similar assessments of DEM performance in other (data-sparse) coastal regions in the world. As elevation forms the basis for any coastal impact assessment, it needs to be handled carefully and with scrutiny, a practice which is currently often lacking in the coastal research community. Similar to sea level, land elevation is not static but dynamic and this should be considered properly in coastal impact assessment. The assessment of land elevation depends on the respective DEM dataset and vertical reference used, as well as the relative SLR and surface elevation change. As new elevation datasets become available, as do vertical reference systems such as geoid models and sea-level datasets, it should be prioritised to provide end users not only with latest available elevation data but also reference them to latest vertical reference systems or sea level, depending on the intended end use. Providing the coastal research community with regular updates of most recent DEMs, already referenced to an actual sea-level datum, such as latest available MDT, will take complex vertical datum conversion steps away from non-specialist end users and thereby reduce overall uncertainties in coastal elevation and elevation-related SLR and other coastal hazard impact assessment.

## Methods

### Updating the vertical datum of local elevation data to continuous local mean sea level

**Fig. 7:**
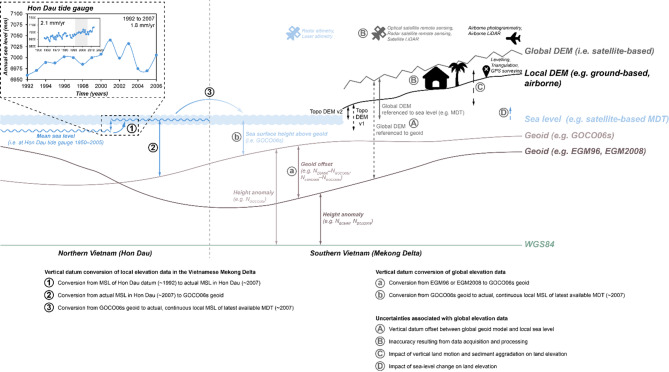
Vertical datums of elevation and sea-level datasets used for the example of the Mekong Delta together with steps of datum conversion for local (1–3) and global elevation data (a–b) and sources of uncertainty associated with global elevation data (A–E). To convert local geodetic heights from mean sea level (MSL) of Hon Dau datum, which is MSL over 1950 to 2005^86,87^, to actual, continuous local MSL as defined by latest available mean dynamic topography (MDT) (1993–2021)^51^, sea-level rise at Hon Dau tide gauge between 1992 (i.e. year for MSL of Hon Dau datum) and 2007 (i.e. year for MSL of MDT time period) had to be considered (step 1)^88^. Subsequent steps of converting the local geodetic heights to MDT included the determination of offset to the global GOCO06s geoid (step 2) and subtraction of MDT data (step 3). To convert global digital elevation models (DEMs) from global geoid models such as EGM96 or EGM2008 to actual, continuous local MSL as defined by MDT, they were first converted to GOCO06s (step a) and then corrected to MDT (step b). Arrows indicate the relations between vertical reference systems that are relevant to convert elevation and sea-level datasets (solid and short-dashed lines). This figure also illustrates the various sources of uncertainty associated with global DEMs in coastal lowlands, which are vertical datum offset (A), inaccuracy (B), and actuality in terms of impacts due to land elevation change (C) and sea-level change (D). Icons of this figure (i.e. satellite, plane and house) were obtained from Font Awesome Free v7.1.0 (CC-BY 4.0 License).

TopoDEM_v2 was generated, similar to Topo DEM v1^38^, using elevation data acquired between 2001 and 2003 and referenced to mean sea level (MSL) defined by Hon Dau tide gauge, which is located ca. 1400 km north of the study area^86^. We applied a vertical datum conversion from MSL as defined by Hon Dau tide gauge to MSL as defined by global-scale, satellite altimetry-based mean dynamic topography (MDT) to reference the locally measured elevation points to MSL of the Mekong Delta coast. To convert the geodetic heights to MSL as defined by latest available MDT (MDT HYBRID-CNES-CLS2022; 1993–2021^51^), they were first corrected for SLR that occurred at Hon Dau since datum establishment which is MSL over 1950 to 2005^86,87^. Consequently, MSL for MDT^51^ dates around April 2007 while it is 1992 for Hon Dau datum. Tide gauge information of Hon Dau obtained from the Permanent Sea Level Stations repository indicates 1.8 mm/yr of SLR and that sea level has risen by 0.027 m between 1992 (i.e. year for MSL of Hon Dau datum) and 2007 (i.e. year for MSL of MDT time period)^88^. We did not include the contribution of vertical land motion to relative sea-level rise as the Hon Dau tide gauge is situated on bedrock and is therefore not subject to (non-linear) vertical land motion stemming from the unconsolidated zone. It may be subject to a low rate of vertical land motion stemming from tectonics but we do not have further information on this. Consequently, subtracting the amount of 0.027 m from the elevation points converts them to actual MSL at Hon Dau (Fig. 7). We assume that this determined actual MSL equalises actual MSL as given by MDT^51^. However, in order to convert the local elevation data to continuous MSL along the coast of the Mekong Delta, we use the difference (= 1.173 m, SD = 0.005 m) between Hon Dau MSL (measured at the tide gauge) and GOCO06s geoid at the tide gauge location and assume the same difference between geoid GOCO06s and local MSL for the Mekong Delta. Our assumption is confirmed as Vung Tau, the tide gauge situated closest to the delta, shows a similar difference between MSL and GOCO06s geoid (= 1.163 m, SD = 0.009 m). Geoid height above the WGS84 ellipsoid was obtained from the openly accessible calculation service of the International Centre for Global Earth Models (ICGEM) at a spatial resolution of 0.085 deg^85^. For each geoid, the obtained point data was interpolated into a raster using multiquadric radial basis functions which showed the best interpolation performance. To be comparable to the spatial resolution of the digital elevation models (DEMs) used in this study, the geoid rasters were resampled to 90 m × 90 m and 1000 m × 1000 m resolution using bilinear resampling. Being suitable for continuous surfaces/rasters, the bilinear resampling algorithm reveals a good compromise between accuracy and smoothness and therefore a more realistic representation of terrain morphology than other resampling algorithms, especially useful for hydraulic applications (ref^89^ and references therein). Geoid offsets were calculated based on subtraction of the resampled geoid rasters.

Given that the quality of satellite altimetry measurements along the coast may be susceptible to land contamination and limitations due to geophysical and environmental corrections^83,84,90^, we do not apply an individual MDT value but apply a radius of 100 km. This threshold value is supported by ref^91^, who document the decrease in accuracy of satellite altimetry to occur within 100 km from the coast. Consequently, we add the average offset of 1.173 m to reference all elevation points to the GOCO06s geoid. Next, they were converted to continuous local MSL as represented by MDT HYBRID-CNES-CLS2022 by subtracting MDT^51^ and following the updated approach of Seeger et al.^11^. To avoid introducing artefacts due to the differential spatial resolution of MDT and elevation data and to enable datum conversion to MDT not only for the most coastal point elevations but also inland, we resampled the MDT dataset to a resolution of 90 m using bilinear resampling and extrapolated the data up to 500 km inland. For this, we used an Inverse Distance Weighting algorithm and applied a smoothing factor of 0.5 which provides the best compromise in smoothing the extrapolation while keeping fidelity to the data. The distance threshold was chosen to provide the best compromise in covering vast coastal lowlands (thereby also including the largest delta in the world, i.e. the Ganges-Brahmaputra-Meghna Delta), while also keeping sufficient quality in face of decreasing extrapolation performance with increasing distance. To identify the most suitable interpolation algorithm, we tested several interpolation algorithms (Inverse Distance Weighting with a standard neighbourhood type, Inverse Distance Weighting with a smooth neighbourhood type, Empirical Bayesian Kriging with empirical transformation and standard circular neighbourhood type, multiquadric radial basis functions with standard neighbourhood type) and evaluated their differences by investigating the respective error metrices. As these were negligible, we employed Inverse Distance Weighting, following the approach of refs^33,34^, which puts greater weight to the most proximal MDT values. The resampled and extrapolated MDT dataset was subtracted from the pre-processed elevation measurements and therewith concluded the datum conversion process to actual, continuous local MSL along the Mekong Delta coast.

TopoDEM_v2 was generated through a two-phased interpolation using Empirical Bayesian Kriging with empirical transformation and exponential modelling within the Geostatistical Wizard in the ArcGIS Pro Analysis 3.1.3 environment. The Empirical Bayesian Kriging algorithm, transformation and modelling settings were chosen based on extensive tests of interpolation methods and validations by local elevation points by Minderhoud et al.^38^. Firstly, the DEM for the delta plain was interpolated after excluding elevations > 10 m to ensure high DEM accuracy for the delta plain and minimise the risk of higher elevations from outcrops impacting DEM interpolation in the flat, low-lying surroundings (similar to Topo DEM v1^38^). Secondly, the entire area was interpolated with no data exclusion. Subsequently, the extent of the delta plain was clipped from the first interpolation to replace the elevation data in the second interpolation, thereby ensuring the best delta plain representation. TopoDEM_v2 has a spatial resolution of 500 m × 500 m, justified by the data density per km^292^ and to facilitate proper DEM comparison for the delta plain, all elevations > 10 m, outcrops and water bodies were masked following the same procedure as described in Topo DEM v1^38^.

### Assessment of global elevation model performance

We revisit land elevation in the Vietnamese Mekong Delta by extending the assessment of DEM performance, integrate many more elevation datasets than previous works, updating them to latest available MSL, and widening the spatial coverage to include also surrounding provinces. Global DEMs are often used to overcome the limited availability or absence of local elevation data or its coarse spatial resolution impacting the precision of follow-up applications. However, as the low accuracy of global DEMs is often not or only inadequately addressed, we assessed the quality for the majority of open and freely available DEMs in the Vietnamese Mekong Delta and surrounding provinces, quantifying both respective DEM accuracy and the impact of absent or incomplete and incorrect vertical datum conversion.

In total, 11 global DEMs were used for the comparison with Topo DEM v1 and v2, namely SRTM^20^ (original spatial resolution: 30 m), ACE2^25–27^ (original spatial resolution: 30 m), MERITDEM^29^ (original spatial resolution: 90 m), ASTGTM v003^21,22^ (original spatial resolution: 30 m), AW3D30^23^ (original spatial resolution: 30 m), TanDEM-X 90m^93^, Copernicus DEM^28^ (original spatial resolution: 30 m), FABDEM v1.0^30^ (original spatial resolution: 30 m), CoastalDEM v2.1^31,32^ (original spatial resolution: 90 m), GLL-DTM v2^34^ (original spatial resolution: ~1000 m), DeltaDTM^35^ (original spatial resolution: 30 m). Refs^20–23,25–32,34,35,93^ also provide information on the respective DEM’s global vertical error statistics, which may differ from those in the Mekong Delta. While both SRTM and MERITDEM have been addressed by studies of Minderhoud et al.^38^, the quantified uncertainties could only be attributed tentatively. More recent DEMs such as FABDEM have been applied in the Mekong Delta (e.g., ref^73^), however without assessing their quality. To quantify also the impacts of incomplete vertical datum conversion and difference in elevation threshold applied in DEM interpolation, we integrate two versions of the GLL-DTM v2, the first referenced to MDT (CNES-CLS13 MDT^94^) as performed by Vernimmen and Hooijer^34^ and a second following the approach of this study, as well as both DeltaDTM v1 and v1.1^35,52^.

Our quality assessments included both validation with the local TopoDEM_v2 to assess and quantify spatial patterns and differences, as well as spot height comparison with elevation at locations of point measurements that fed the local DEM interpolation. Beforehand, the global DEMs were pre-processed. Single DEM tiles covering the area of interest were mosaicked and projected to UTM 48 N based on the WGS84 ellipsoid. Subsequently, the vertical datums of the DEMs (i.e. EGM96 and EGM2008) were converted to MSL as defined by MDT^51^ and considering to correct for respective geoid offsets. Although GLL-DTM v2 is already provided with a reference to MSL^34^, we obtained the DEM referenced to EGM96 and could therefore correct for the previously unconsidered geoid offset by applying the same workflow as for the other global DEMs (Fig. 7). All DEMs, including versions with the vertical reference as provided in the respective data repositories as well as with respect to MSL (this study), were masked by applying their respective water body masks. For ACE2, MERITDEM and CoastalDEM v2.1 and FABDEM v1.0, water bodies were excluded based on masks from the underlying source data, which is SRTM and only in case of FABDEM Copernicus. GLL-DTM v2 and DeltaDTM v1 and v1.1 did not require this processing step as the spatial resolution of GLL-DTM v2 is too coarse to adequately resolve the deltaic river and channel network while in DeltaDTM, these values are already eliminated. Largely negative and therefore likely erroneous elevation values were excluded by applying a threshold of <−7 m above the respective vertical datum^11,33,34^. As for Topo DEM v 1 and v2, we applied the same outcrop mask to the global DEMs. In addition, all global DEMs were resampled to 500 m × 500 m spatial resolution using bilinear resampling and snapping to the TopoDEMs’ extent. Only for GLL-DTM v2 with a resolution of ~ 1000 m × ~ 1000 m, we resampled the local TopoDEM_v2 using bilinear resampling to match the same spatial resolution as GLL-DTM v2. All these pre-processing steps enable a proper conduction of DEM comparison in terms of difference mapping with the local TopoDEM_v2 and ensure comparability. Only for extracting point elevations from each DEM and quantifying geoid offset per DEM, the respective spatial resolution of the original DEM was used.

### Quantification of vertical datum offsets

To quantify the overall discrepancies between geoid models and actual continuous local sea-level height along the coast for the entire Mekong Delta, we used geoid information for EGM96, EGM2008 and GOCO06s obtained in form of height anomaly to the WGS84 ellipsoid from the ICGEM calculation service^85^. The respective offsets between EGM96 and EGM2008 geoids to GOCO06s were calculated and subsequently subtracted from mean dynamic topography, i.e. MDT – (EGM–GOCO06s). As the processing of the geoid rasters included bilinear resampling to 90 m spatial resolution, all statistics for the respective offsets of EGM96 and EGM2008 to MSL are given at 90 m × 90 m spatial resolution. However, in order to account for an adequate vertical datum conversion of GLL-DTM v2 and compare it to the original GLL-DTM v2^34^, which serves as an example of incomplete vertical datum conversion, we used EGM96 geoid data at 1000 m × 1000 m spatial resolution. Consequently, the resulting statistics are provided at the same resolution.

### Quantifying sources of uncertainties in elevation assessment

To assess the impact of data-inherent inaccuracy, vertical datum offset and data actuality on the quality of elevation datasets, we include the root mean square errors with TopoDEM_v2 determined for the DEMs referenced to their original datum in the Mekong Delta as well as the amount by which the error reduced after the vertical datum conversion to MDT-based MSL. This value reflects the uncertainty related to vertical datum offset. As the uncertainty related to data actuality depends on relative sea-level change over time, we include both land subsidence and SLR, by using simulated, non-linear extraction-induced land subsidence^56,57^ (B1 scenario of Minderhoud et al.^57^) and annual rates of local, delta-average SLR estimated from PSMSL data of Vung Tau tide gauge^58^ and IPCC AR6 total rates of sea-level change^59–61^ (Supplementary Table 6). The beginning of the time period, over which absolute sea-level change and extraction-induced land subsidence were integrated, depends on the time of data acquisition of the individual DEM, while the end of the time period is determined by the 50th percentile of the sea-level reference period (i.e. 2007; Fig. 5a). Subtracting the quantified uncertainties of vertical datum offset, extraction-induced land subsidence and absolute sea-level change from the root mean square errors of the respective DEMs referenced to their respective original vertical datum allowed to quantify the remaining uncertainty that can be attributed to DEM acquisition and processing issues. Including relative sea-level change after 2007 allows to update the elevation uncertainty assessment to the present time (i.e. 2025; Fig. 5b) and results in increased root mean square errors.

## Supplementary Information

Below is the link to the electronic supplementary material.

Supplementary Material 1

## Data Availability

The Digital Elevation Models for the Mekong Delta based on global digital elevation models and converted to local mean sea level as indicated by mean dynamic topography (following the approach of this study), are publicly available under a CC-BY-NC-SA 4.0 license here: 10.5281/zenodo.18347786(for the Mekong Delta only); and here: 10.5281/zenodo.18347901 (for the Mekong and its surroundings). TopoDEM_v2 is publicly available under a CC-BY 4.0 license here: 10.5281/zenodo.1834609.
