# Supplementary material for: Elevation uncertainties in the Mekong Delta quantified using a transferable approach
Source: Sci Rep. 2026 Feb 4;16:4993. doi: 10.1038/s41598-026-38315-y (PMC12876989; doi:10.1038/s41598-026-38315-y)
Supplement: Supplementary file 1 — Supplementary Material 1 [file 41598_2026_38315_MOESM1_ESM.pdf]

Elevation uncertainties in the Mekong Delta quantified using a transferable approach

Katharina Seeger[1,2,3*], and Philip S.J. Minderhoud[1,3,4]

[1]Soil Geography and Landscape Group, Wageningen University & Research; Wageningen, NL-6708PB, Netherlands
[2]Institute of Geography, University of Cologne; Cologne, 50923, Germany
[3]Department of Civil, Environmental and Architectural Engineering, University of Padova; Padova, Italy
[4]Department of Subsurface and Groundwater Systems, Deltares Research Institute; Utrecht, The Netherlands
[*]katharina.seeger@wur.nl, katharina.seeger@unipd.it, k.seeger@uni.koeln.de

Supplementary Information

Performance of digital elevation models in the Vietnamese Mekong Delta

Which one is the best? – On the local validation of global satellite-based digital elevation models: Comparison with local point elevations

Extracting local point elevations from the respective global DEMs in form of a spot height comparison reveals similar results as the spatial comparison with TopoDEM_v2 and yields only slight changes in the error and height residual statistics. For most of the DEMs which are comparably higher than indicated by local elevation, error metrics lower by ca. 2–5 cm compared to the statistics of the comparison to TopoDEM_v2 (Table 2; Supplementary Table 1). For those DEMs that perform already well in comparison with TopoDEM_v2 (i.e. DeltaDTM) or are on average consistent with the local data (i.e. CoastalDEM v2.1), pointwise determined average errors slightly increase and height residuals become (more) negative while for GLL-DTM v2, at least mean error reduces further. This pattern holds true for the comparison with low-lying spot heights (i.e. ≤10 m) as well (Table 2; Supplementary Table 2). Probably the most remarkable difference between DEM spatial comparison and validation by local point elevations is the decrease of median height residual for SRTM by 13–14 cm to 0.04 m for point elevation comparison with all and only low-elevation spot heights, respectively (Supplementary Tables 1 and 2). However, we do not consider these slight changes as significant but rather explain them by respective capacities and limitations to resolve local point settings. Instead, we interpret the results of this point elevation comparison being similar to outcomes of the spatial comparison with TopoDEM_v2 and to strengthen our overall finding that among the assessed DEMs, most recently processed coastal elevation data provides the most accurate results, of which we strongly recommend the usage of DeltaDTM v1 referenced to local continuous, actual MSL, which shows the overall best agreement with local elevation data in the Vietnamese Mekong Delta.

DEM	N	Mean spot (m)	Median spot (m)	Min. spot (m)	Max. spot (m)	Mean DEM (m)	Median DEM (m)	Min. DEM (m)	Max. DEM (m)	σ DEM (m)	Max. negative HR (m)	Max. positive HR (m)	Mean error (m)	MAE (m)	Median error (m)	RMSE (m)
TopoDEM_v2	18447	0.79	0.66	-1.06	9.95	0.77	0.70	-0.34	7.58	0.60	2.46	-4.76	-0.03	0.13	0.01	0.26
Topo DEM v1	18685	0.80	0.67	-1.06	9.95	0.82	0.74	-0.49	6.91	0.60	-9.25	1.10	-0.03	0.13	0.01	0.26
$SRTM_{MDT}$	18865	0.83	0.67	-1.06	99.95	0.98	0.83	-6.75	72.99	2.55	-52.76	54.18	0.15	1.85	0.04	2.52
$SRTM_{EGM96}$	18997	0.83	0.67	-1.06	99.95	2.21	2.04	-6.72	74.46	2.60	-51.63	55.24	1.38	2.17	1.28	2.89
$ACE2_{MDT}$	16809	0.80	0.66	-1.06	99.95	0.97	0.86	-5.98	80.84	1.87	-26.38	24.26	0.17	1.24	0.11	1.65
$ACE2_{EGM96}$	16853	0.80	0.66	-1.06	99.95	2.23	2.12	-5.11	82.31	1.94	-25.23	25.19	1.44	1.75	1.38	2.21
$MERITDEM_{MDT}$	18365	0.80	0.66	-1.06	99.95	2.02	1.94	-3.32	79.04	1.26	-24.13	26.85	1.22	1.35	1.24	1.60
$MERITDEM_{EGM96}$	18367	0.80	0.66	-1.06	99.95	3.29	3.25	-5.15	80.51	1.35	28.09	-22.98	2.49	2.52	2.52	2.72
ASTGTM $v003_{MDT}$	19028	0.83	0.67	-0.45	99.95	8.60	7.46	-1.23	80.07	5.08	-53.20	80.22	7.78	7.80	6.63	9.36
ASTGTM $v003_{EGM96}$	19028	0.83	0.67	-0.45	99.95	9.87	8.76	0.00	80.89	5.08	-52.07	81.04	9.04	9.05	7.91	10.42
$AW3D30_{MDT}$	15977	0.84	0.68	-1.06	61.95	1.27	1.02	-6.27	43.07	2.43	-51.17	41.13	0.43	1.73	0.25	2.48
$AW3D30_{EGM96}$	16055	0.84	0.68	-1.06	61.95	2.50	2.22	-6.97	44.14	2.50	-50.04	42.19	1.66	2.19	1.50	2.99
$TanDEM\text{-}X_{MDT}$	18162	0.81	0.66	-1.06	99.95	1.69	1.17	-5.40	81.73	1.82	26.80	-59.40	0.89	1.06	0.41	1.92
$TanDEM\text{-}X_{EGM96}$	18167	0.81	0.66	-1.06	99.95	2.94	2.47	-3.79	83.20	1.84	-58.28	28.05	2.14	2.17	1.68	2.72
Copernicus DEM_{MDT}	16479	0.88	0.75	-0.45	99.95	1.68	1.07	-4.15	62.50	1.98	-48.42	60.56	0.80	1.09	0.22	2.07
Copernicus $DEM_{EGM2008}$	16479	0.88	0.75	-0.45	99.95	2.87	2.26	-2.92	63.55	1.98	-47.32	61.61	1.98	2.01	1.40	2.74
$FABDEM_{MDT}$	14605	0.87	0.75	-0.45	99.95	1.28	1.03	-1.51	80.92	1.30	-19.02	28.25	0.41	0.62	0.23	1.10
$FABDEM_{EGM2008}$	16341	0.88	0.75	-0.45	99.95	2.45	2.18	-0.92	67.74	1.37	-52.92	43.62	1.57	1.60	1.37	2.00

CoastalDEM v2.1$_{MDT}$	18961	0.83	0.67	-1.06	99.95	0.75	0.78	-2.46	69.86	1.26	-60.23	36.45	-0.08	0.82	-0.02	1.25
CoastalDEM v2.1$_{EGM96}$	18940	0.83	0.67	-1.06	99.95	2.02	2.00	-1.15	53.92	1.29	-58.87	51.47	1.20	1.37	1.20	1.78
GLL-DTM v2$_{MDT (this study)}$	17346	0.76	0.66	-0.45	9.95	0.81	0.75	-0.51	7.02	0.55	-8.47	4.12	0.05	0.33	0.09	0.46
GLL-DTM v2$_{MDT (original)}$	17354	0.76	0.66	-0.45	7.98	0.95	0.89	-0.75	7.19	0.68	-7.51	4.19	0.19	0.42	0.26	0.55
DeltaDTM v1$_{MDT}$	14055	0.86	0.75	-0.45	7.98	0.75	0.68	-1.88	6.77	0.61	-7.35	4.71	-0.11	0.36	-0.11	0.52
DeltaDTM v1$_{EGM2008}$	16479	0.86	0.75	-0.45	9.95	1.94	1.84	-0.98	9.77	0.70	-7.09	7.20	1.07	1.11	1.11	1.21
DeltaDTM v1.1$_{MDT}$	16462	0.87	0.75	-0.45	25.95	0.63	0.53	-3.55	28.95	0.77	-25.09	27.01	-0.24	0.40	-0.19	0.67
DeltaDTM v1.1$_{EGM2008}$	16462	0.87	0.75	-0.45	25.95	1.81	1.70	-2.32	30.00	0.79	-23.98	28.06	0.94	0.99	1.00	1.13

Supplementary Table 1. Performance of local and global DEMs in the Vietnamese Mekong Delta validated by local point elevations for versions referenced to mean dynamic topography (MDT) and their original vertical reference. N – number of spot heights in the study area, with no-data values excluded for each DEM, respectively; Mean spot – mean point elevation in the study area; Median spot – median point elevation in the study area; Min. spot – minimum point elevation in the study area; Max. spot – maximum point elevation in the study area; Mean DEM – mean DEM elevation in the study area; Median DEM – median DEM elevation in the study area; Min. DEM – minimum DEM elevation in the study area; Max. DEM – maximum DEM elevation in the study area; σ DEM – standard deviation of DEM elevation in the study area; HR – height residual; MAE – mean absolute error; RMSE – root mean square error.

DEM	N	Mean spot (m)	Median spot (m)	Min. spot (m)	Max. spot (m)	Mean DEM (m)	Median DEM (m)	Min. DEM (m)	Max. DEM (m)	σ DEM (m)	Max. negative HR (m)	Max. positive HR (m)	Mean error (m)	MAE (m)	Median error (m)	RMSE (m)
TopoDEM_v2	18447	0.79	0.66	-1.06	9.95	0.77	0.70	-0.34	7.58	0.60	-4.76	2.46	-0.03	0.13	0.01	0.26
Topo DEM v1	18685	0.80	0.67	-1.06	9.95	0.82	0.74	-0.49	6.91	0.60	-9.25	1.10	-0.03	0.13	0.01	0.26
SRTM$_{MDT}$	18858	0.82	0.67	-1.06	9.95	0.98	0.82	-6.75	56.12	2.49	-9.83	54.18	0.16	1.84	0.04	2.48
SRTM$_{EGM96}$	18990	0.82	0.67	-1.06	9.95	2.20	2.04	-6.72	57.19	2.55	-8.33	55.24	1.39	2.16	1.28	2.83
ACE2$_{MDT}$	16806	0.79	0.66	-1.06	9.95	0.96	0.86	-5.98	25.10	1.76	-6.98	24.26	0.17	1.24	0.11	1.63
ACE2$_{EGM96}$	16850	0.79	0.66	-1.06	9.95	2.23	2.12	-5.11	26.04	1.83	-5.42	25.19	1.44	1.75	1.38	2.20
MERITDEM$_{MDT}$	18362	0.79	0.66	-1.06	9.95	2.02	1.94	-3.32	30.80	1.10	-5.37	26.85	1.22	1.34	1.25	1.58
MERITDEM$_{EGM96}$	18364	0.79	0.66	-1.06	9.95	3.28	3.25	-5.15	32.04	1.20	-6.12	28.09	2.49	2.52	2.52	2.70
ASTGTM v003$_{MDT}$	19023	0.82	0.67	-0.45	9.95	8.60	7.46	-1.23	80.07	5.06	-4.44	80.22	7.79	7.79	6.63	9.35
ASTGTM v003$_{EGM96}$	19023	0.82	0.67	-0.45	9.95	9.86	8.76	0.00	80.89	5.06	-3.25	81.04	9.05	9.05	7.91	10.41
AW3D30$_{MDT}$	15971	0.83	0.68	-1.06	9.95	1.26	1.02	-6.27	43.07	2.42	-7.74	41.13	0.44	1.72	0.25	2.41
AW3D30$_{EGM96}$	16049	0.83	0.68	-1.06	9.95	2.50	2.22	-6.97	44.14	2.49	-7.65	42.19	1.67	2.19	1.50	2.95
TanDEM-X$_{MDT}$	18155	0.79	0.66	-1.06	9.95	1.69	1.17	-5.40	29.25	1.72	-6.35	26.80	0.90	1.05	0.41	1.81
TanDEM-X$_{EGM96}$	18160	0.79	0.66	-1.06	9.95	2.94	2.47	-3.79	30.49	1.73	-4.74	28.05	2.15	2.16	1.68	2.65
Copernicus DEM$_{MDT}$	16474	0.87	0.75	-0.45	9.95	1.67	1.07	-4.15	62.50	1.93	-5.17	60.56	0.81	1.08	0.22	2.00
Copernicus DEM$_{EGM2008}$	16474	0.87	0.75	-0.45	9.95	2.86	2.26	-2.92	63.55	1.93	-3.92	61.61	1.99	2.01	1.40	2.69
FABDEM$_{MDT}$	14603	0.86	0.75	-0.45	9.95	1.27	1.03	-1.51	32.19	1.10	-5.92	28.25	0.41	0.61	0.23	1.08
FABDEM$_{EGM2008}$	16336	0.86	0.75	-0.45	9.95	2.44	2.18	-0.92	45.56	1.26	-4.00	43.62	1.58	1.59	1.37	1.93
CoastalDEM v2.1$_{MDT}$	18954	0.81	0.67	-1.06	9.95	0.75	0.78	-2.46	38.90	1.15	-5.89	36.45	-0.07	0.82	-0.02	1.08
CoastalDEM v2.1$_{EGM96}$	18933	0.81	0.67	-1.06	9.95	2.02	2.00	-1.15	53.92	1.23	-6.66	51.47	1.21	1.36	1.28	1.64
GLL-DTM v2$_{MDT (this study)}$	17346	0.76	0.66	-0.45	9.95	0.81	0.75	-0.51	7.02	0.55	-8.47	4.12	0.05	0.33	0.09	0.46
GLL-DTM v2$_{MDT (original)}$	17354	0.76	0.66	-0.45	7.98	0.95	0.89	-0.75	7.19	0.68	-7.51	4.19	0.19	0.42	0.26	0.55
DeltaDTM v1$_{MDT}$	14055	0.86	0.75	-0.45	7.98	0.75	0.68	-1.88	6.77	0.61	-7.35	4.71	-0.11	0.36	-0.07	0.52
DeltaDTM v1$_{EGM2008}$	16479	0.86	0.75	-0.45	9.95	1.93	1.84	-0.98	9.77	0.70	-7.09	7.20	1.07	1.11	1.11	1.21
DeltaDTM v1.1$_{MDT}$	16459	0.86	0.75	-0.45	9.95	0.63	0.53	-3.55	28.95	0.76	-8.60	27.01	-0.24	0.40	-0.19	0.64
DeltaDTM v1.1$_{EGM2008}$	16459	0.86	0.75	-0.45	9.95	1.81	1.70	-2.32	30.00	0.77	-7.50	28.06	0.94	0.99	1.00	1.11

Supplementary Table 2. Performance of local and global DEMs in the Vietnamese Mekong Delta validated by local point elevations ≤10 m for versions referenced to mean dynamic topography (MDT) and their original vertical reference. N – number of spot heights in the study area, with no-data values excluded for each DEM, respectively; Mean spot – mean point elevation in the study area; Median spot – median point elevation in the study area; Min. spot – minimum point elevation in the study area; Max. spot – maximum point elevation in the study area; Mean DEM – mean DEM elevation in the study area; Median DEM – median DEM elevation in the study area; Min. DEM – minimum DEM elevation in the study area; Max. DEM – maximum DEM elevation in the study area; σ DEM – standard deviation of DEM elevation in the study area; HR – height residual; MAE – mean absolute error; RMSE – root mean square error.

Converting vertical datum to actual continuous local sea level

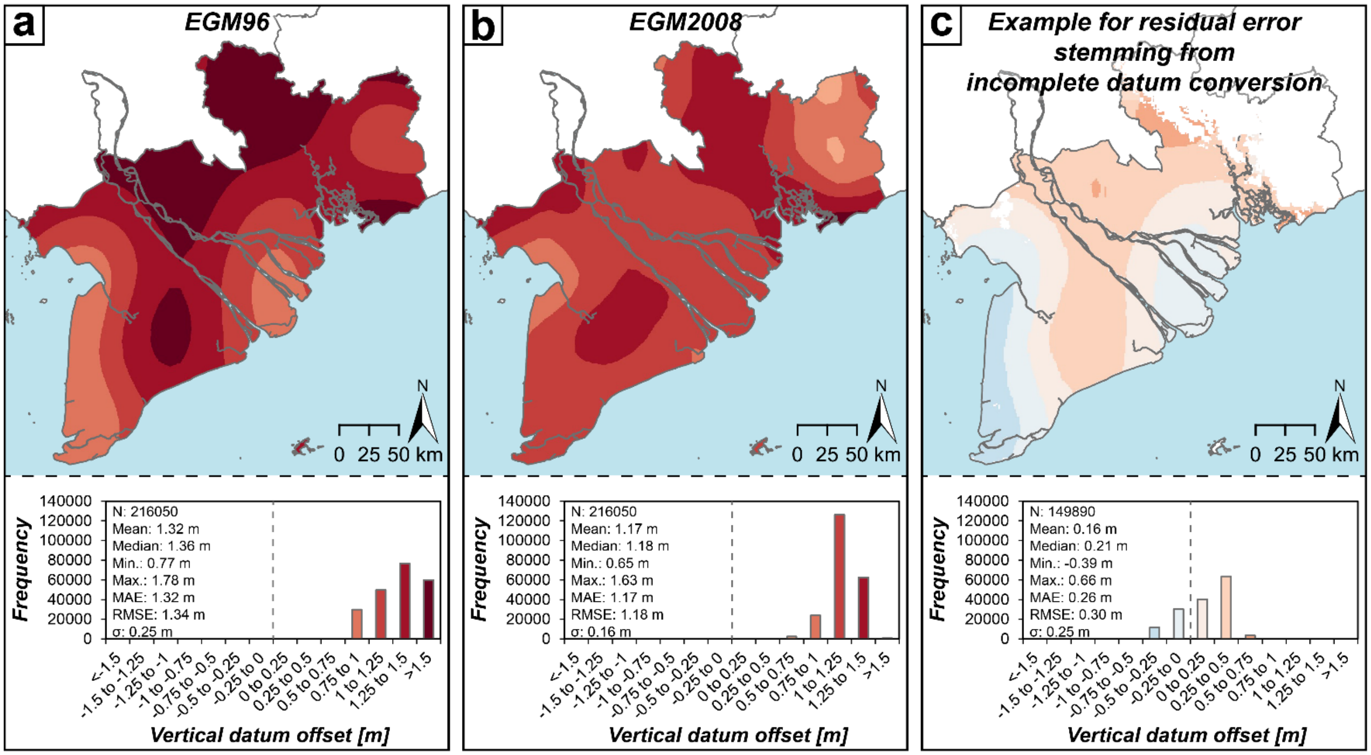

Supplementary Figure 1. Offsets between commonly used geoid models (EGM96 (a); EGM2008 (b)) as well as an example of incomplete datum conversion (c) and local mean sea level as indicated by mean dynamic topography of Jousset et al.[1] in the Vietnamese Mekong Delta. Sea-level heights above EGM96 and EGM2008 were obtained by applying a conversion of $MDT_{GOCO06s}$ – (EGM96 – GOCO06s) and $MDT_{GOCO06s}$ – (EGM2008 – GOCO06s). The residual error arising from incomplete datum conversion was calculated for the example of GLL-DTM v2[2] by subtracting the GLL-DTM v2 corrected to MDT (this study) from GLL-DTM v2 referenced to its original vertical datum[2].

Performance of digital elevation models in the Vietnamese Mekong Delta and its adjacent provinces

Which one is the best? – On the local validation of global satellite-based digital elevation models: Comparison with local topographical digital elevation model

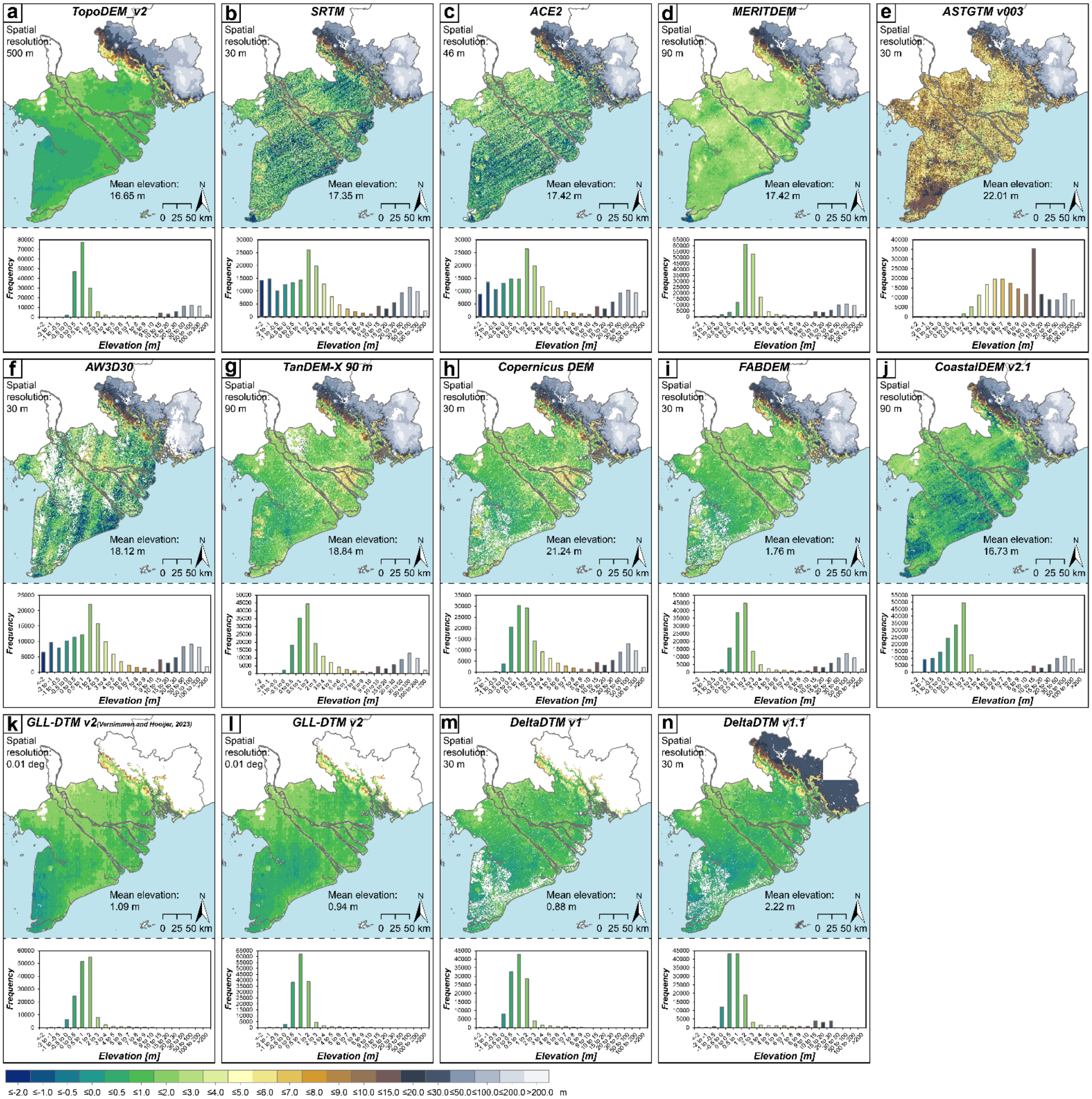

Supplementary Figure 2. Digital elevation models for the Vietnamese Mekong Delta and its adjacent provinces, all referenced to actual, local continuous mean sea level as indicated by mean dynamic topography of Jousset et al.[1].

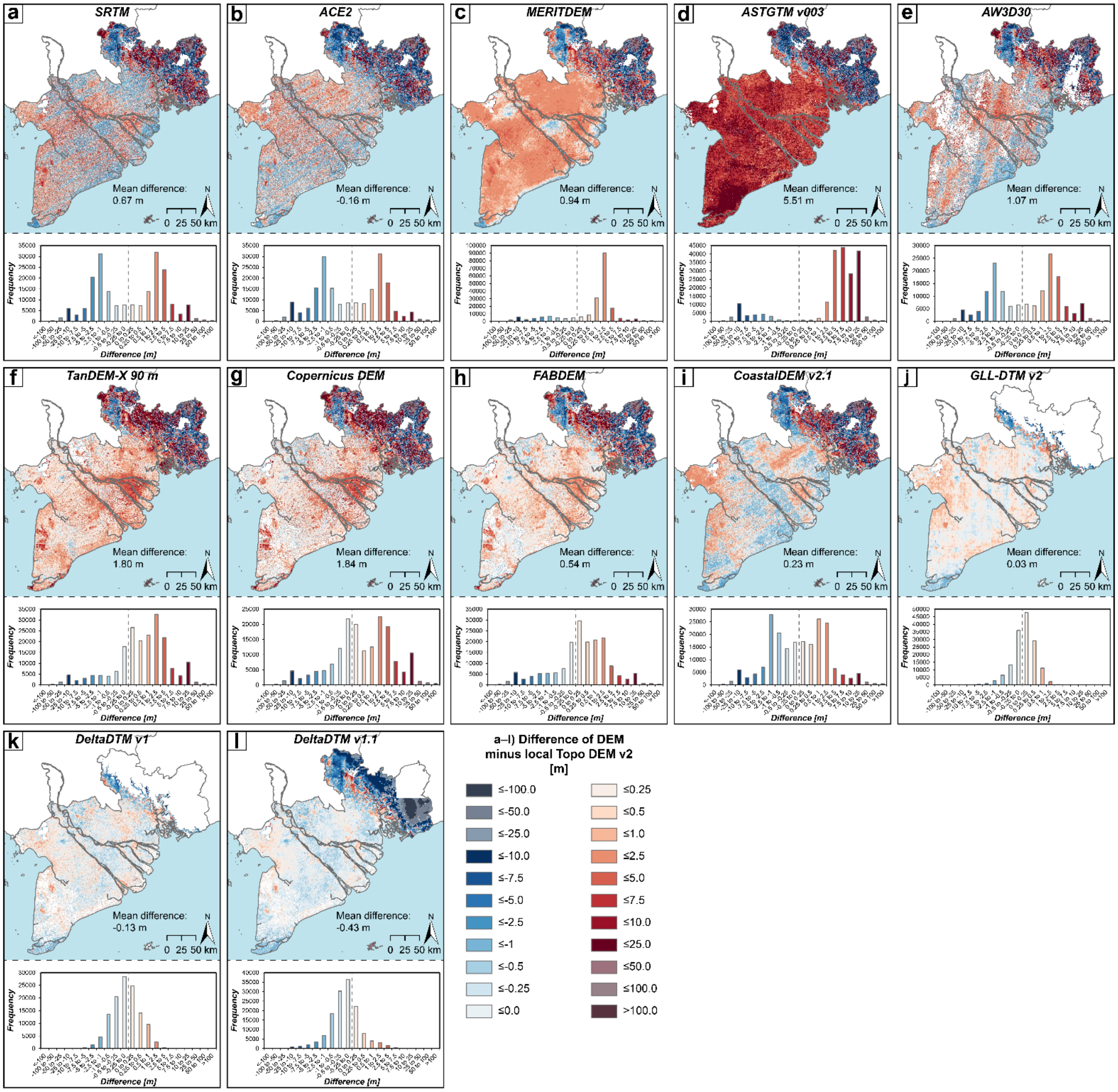

Supplementary Figure 3. Height residuals of DEMs referenced to mean sea level compared to TopoDEM_v2 for the Vietnamese Mekong Delta and its adjacent provinces.

DEM	N	Mean DEM (m)	Median DEM (m)	Min. DEM (m)	Max. DEM (m)	σ DEM (m)	Max. negative HR (m)	Max. positive HR (m)	Mean error (m)	MAE (m)	Median error (m)	RMSE (m)	Difference in RMSE (m)	Discrepancy (%)
TopoDEM_v2	219365	16.65	0.91	-0.65	268.65	38.64								
SRTM$_{MDT}$	203572	17.35	1.86	-6.95	922.24	42.75	-192.81	665.65	0.67	4.34	0.14	12.16	0.11	0.9
SRTM$_{EGM96}$	205265	18.51	3.00	-7.00	924.00	42.63	-190.72	667.41	1.97	4.63	1.39	12.27		
ACE2$_{MDT}$	192353	17.42	1.77	-6.96	869.17	42.86	-166.59	620.86	-0.16	4.22	-0.06	12.28	0.04	0.4
ACE2$_{EGM96}$	192702	18.71	3.10	-7.00	870.88	42.84	-164.94	622.57	1.16	4.47	1.21	12.32		
MERITDEM$_{MDT}$	205967	17.42	2.35	-5.13	936.53	40.99	-183.97	679.93	0.94	3.57	1.20	11.42	0.19	1.6
MERITDEM$_{EGM96}$	205968	18.74	3.70	-3.87	938.24	41.01	-182.30	681.64	2.26	4.46	2.48	11.60		
ASTGTM v003$_{MDT}$	205492	22.01	8.74	-1.46	916.55	39.57	-202.94	659.95	5.51	9.05	5.80	14.60	0.53	3.5
ASTGTM v003$_{EGM96}$	205492	23.33	10.00	0.00	918.00	39.58	-201.72	661.41	6.82	9.96	7.09	15.12		
AW3D30$_{MDT}$	159639	18.12	2.00	-6.97	715.51	42.40	-158.80	467.18	1.07	4.33	0.29	11.11	0.20	1.7
AW3D30$_{EGM96}$	160522	19.34	3.00	-7.00	717.00	42.33	-157.11	468.67	2.38	4.73	1.58	11.30		
TanDEM-X$_{MDT}$	197363	18.84	1.92	-6.12	1112.77	43.04	-189.93	1039.27	1.80	3.93	0.56	12.52	0.27	2.1
TanDEM-X$_{EGM96}$	197403	20.15	3.24	-6.53	1113.96	43.06	-188.27	1040.46	3.12	4.88	1.84	12.78		
Copernicus DEM$_{MDT}$	171963	21.40	2.10	-5.05	909.07	45.62	-186.97	652.48	1.84	4.38	0.33	13.20	0.21	1.6
Copernicus DEM$_{EGM2008}$	171963	22.41	3.32	-3.85	910.38	45.54	-185.36	653.79	3.01	5.15	1.53	13.41		
FABDEM$_{MDT}$	172376	19.95	1.51	-2.28	868.39	44.04	-186.92	611.79	0.54	3.54	0.24	12.09	0.59	4.7
FABDEM$_{EGM2008}$	171087	21.21	2.71	-1.72	922.47	44.21	-202.82	665.87	1.72	4.46	1.39	12.68		
CoastalDEM v2.1$_{MDT}$	205653	16.73	1.19	-4.76	937.95	42.32	-208.22	681.36	0.23	3.31	0.01	11.85	0.10	0.9
CoastalDEM v2.1$_{EGM96}$	205655	18.05	2.45	-6.33	939.66	42.34	-206.56	683.07	1.55	3.77	1.31	11.96		
GLL-DTM v2$_{MDT (this study)}$	150580	0.94	0.79	-1.62	9.61	0.88	-9.29	6.29	0.03	0.34	0.08	0.63	0.04	6.1
GLL-DTM v2$_{MDT (original)}$	150982	1.09	0.94	-1.55	9.90	0.99	-8.80	6.36	0.18	0.44	0.25	0.68		
DeltaDTM v1$_{MDT}$	120367	0.88	0.78	-3.11	8.91	0.97	-9.44	7.43	-0.13	0.45	-0.08	0.79	0.52	39.7
DeltaDTM v1$_{EGM2008}$	125276	2.06	1.88	-1.55	9.99	0.99	-8.10	9.03	1.06	1.17	1.11	1.30		
DeltaDTM v1.1$_{MDT}$	139211	2.22	0.62	-5.11	29.15	5.00	-24.95	28.45	-0.43	0.79	-0.21	1.88	0.10	5.2
DeltaDTM v1.1$_{EGM2008}$	139211	3.42	1.79	-4.44	30.00	5.03	-23.87	29.30	0.78	1.33	0.99	1.99		

Supplementary Table 3. Performance of local and global DEMs in the Vietnamese Mekong Delta and its adjacent provinces validated by the local TopoDEM_v2 and DEM-specific vertical datum offsets quantified as differences in RMSE and discrepancies from RMSE of DEMs referenced to mean dynamic topography (MDT). The statistics were extracted from DEMs masked for water bodies and outcrops and resampled to 500 m × 500 m spatial resolution. Discrepancy was calculated as RMSE of DEM$_{MDT}$ minus RMSE of DEM$_{original\ datum}$, divided by RMSE of DEM$_{MDT}$ and expressed in %. N – number of grid cells in the study area, with no-data values excluded for each DEM, respectively; Mean DEM – mean DEM elevation in the study area; Median DEM – median DEM elevation in the study area; Min. DEM – minimum DEM elevation in the study area; Max. DEM – maximum DEM elevation in the study area; σ DEM – standard deviation of DEM elevation in the study area; HR – height residual; MAE – mean absolute error; RMSE – root mean square error.

DEM	N	Mean spot (m)	Median spot (m)	Min. spot (m)	Max. spot (m)	Mean DEM (m)	Median DEM (m)	Min. DEM (m)	Max. DEM (m)	σ DEM (m)	Max. negative HR (m)	Max. positive HR (m)	Mean error (m)	MAE (m)	Median error (m)	RMSE (m)
TopoDEM_v2	27032	14.51	0.95	-1.06	250.00	14.34	0.92	-0.32	254.65	34.73	-124.55	84.10	-0.17	1.35	0.01	5.08
SRTM$_{MDT}$	27242	14.44	0.95	-1.06	250.00	14.86	1.75	-6.75	771.08	38.51	-211.19	521.10	0.42	3.66	0.07	11.63
SRTM$_{EGM96}$	27385	14.37	0.95	-1.06	250.00	16.08	3.00	-6.72	772.78	38.45	-209.52	522.81	1.71	3.93	1.34	11.73
ACE2$_{MDT}$	26129	15.00	0.95	-1.06	250.00	14.72	1.57	-6.85	775.88	38.87	-172.74	525.90	-0.28	3.27	-0.25	11.49
ACE2$_{EGM96}$	26165	14.98	0.95	-1.06	250.00	16.03	2.89	-6.70	777.59	38.86	-171.08	527.61	1.05	3.44	1.03	11.53
MERITDEM$_{MDT}$	27283	14.35	0.95	-1.06	250.00	15.14	2.31	-5.50	786.97	37.28	-210.40	537.00	0.79	2.95	1.14	11.45
MERITDEM$_{EGM96}$	27284	14.35	0.95	-1.06	250.00	16.47	3.68	-4.17	788.68	37.29	-208.73	538.70	2.12	3.86	2.42	11.61
ASTGTM v003$_{MDT}$	27393	14.34	0.95	-0.45	250.00	19.81	8.62	-1.23	789.84	35.95	-226.02	539.87	5.47	8.40	5.69	14.34
ASTGTM v003$_{EGM96}$	27393	14.34	0.95	-0.45	250.00	21.14	9.95	0.00	791.55	35.95	-224.35	541.57	6.80	9.33	7.00	14.87
AW3D30$_{MDT}$	23189	13.56	0.95	-1.06	250.00	14.23	1.89	-6.27	721.66	35.27	-115.68	471.68	0.67	3.43	0.30	9.27
AW3D30$_{EGM96}$	23272	13.52	0.95	-1.06	250.00	15.50	3.15	-6.97	723.36	35.25	-113.91	473.39	1.98	3.84	1.59	9.45
TanDEM-X$_{MDT}$	26439	14.74	0.95	-1.06	250.00	16.41	1.82	-5.40	779.34	39.11	-209.26	529.36	1.66	3.25	0.51	11.94
TanDEM-X$_{EGM96}$	26444	14.74	0.95	-1.06	250.00	17.73	3.16	-3.79	781.05	39.13	-207.60	531.07	2.98	4.23	1.81	12.20
Copernicus DEM$_{MDT}$	24642	15.81	0.95	-0.45	250.00	17.38	1.87	-4.15	770.62	40.13	-216.94	520.65	1.57	3.47	0.32	12.37
Copernicus DEM$_{EGM2008}$	24642	15.81	0.95	-0.45	250.00	18.56	3.08	-2.92	771.93	40.06	-215.33	521.95	2.75	4.25	1.52	12.58
FABDEM$_{MDT}$	22263	16.91	0.96	-0.45	250.00	17.17	1.48	-0.97	743.02	39.55	-205.50	493.04	0.26	2.87	0.23	11.74
FABDEM$_{EGM2008}$	24489	15.91	0.95	-0.45	250.00	17.49	2.65	-0.92	781.64	39.01	-220.48	531.67	1.58	3.61	1.37	12.22
CoastalDEM v2.1$_{MDT}$	27262	14.36	0.95	-1.06	250.00	14.29	1.21	-4.08	782.29	38.07	-226.40	532.32	-0.07	2.78	-0.00	11.77
CoastalDEM v2.1$_{EGM96}$	27262	14.36	0.95	-1.06	250.00	15.62	2.48	-2.39	784.00	38.08	-224.74	534.02	1.26	3.22	1.31	11.84
GLL-DTM v2$_{MDT (this study)}$	19250	1.00	0.68	-0.45	9.99	0.97	0.79	-1.78	9.61	0.96	-9.39	6.10	-0.02	0.45	0.08	0.83
GLL-DTM v2$_{MDT (original)}$	19311	0.99	0.68	-0.45	9.99	1.14	0.95	-1.40	9.88	1.07	-8.90	6.55	0.14	0.53	0.25	0.84
DeltaDTM v1$_{MDT}$	15681	1.12	0.78	-0.45	9.99	0.93	0.72	-1.43	8.44	1.02	-8.94	4.99	-0.18	0.48	-0.07	0.86
DeltaDTM v1$_{EGM2008}$	18704	1.13	0.78	-0.45	9.99	2.15	1.90	-0.98	9.87	1.16	-7.58	7.65	1.02	1.18	1.10	1.35
DeltaDTM v1.1$_{MDT}$	20674	2.79	0.87	-0.45	29.99	2.31	0.64	-3.55	29.09	5.06	-27.47	26.45	-0.48	0.83	-0.22	1.84
DeltaDTM v1.1$_{EGM2008}$	20674	2.79	0.87	-0.45	29.99	3.51	1.82	-2.32	30.00	5.09	-26.39	28.02	0.73	1.30	0.98	1.91

Supplementary Table 4. Performance of local and global DEMs in the Vietnamese Mekong Delta and its adjacent provinces validated by local point elevations for versions referenced to mean dynamic topography (MDT) and their original vertical reference. N – number of spot heights in the study area, with no-data values excluded for each DEM, respectively; Mean spot – mean point elevation in the study area; Median spot – median point elevation in the study area; Min. spot – minimum point elevation in the study area; Max. spot – maximum point elevation in the study area; Mean DEM – mean DEM elevation in the study area; Median DEM – median DEM elevation in the study area; Min. DEM – minimum DEM elevation in the study area; Max. DEM – maximum DEM elevation in the study area; σ DEM – standard deviation of DEM elevation in the study area; HR – height residual; MAE – mean absolute error; RMSE – root mean square error.

DEM	N	Mean spot (m)	Median spot (m)	Min. spot (m)	Max. spot (m)	Mean DEM (m)	Median DEM (m)	Min. DEM (m)	Max. DEM (m)	σ DEM (m)	Max. negative HR (m)	Max. positive HR (m)	Mean error (m)	MAE (m)	Median error (m)	RMSE (m)
TopoDEM_v2	21277	1.13	0.76	-1.06	9.99	1.20	0.77	-0.32	75.71	2.37	-8.75	75.73	0.07	0.30	0.01	1.83
SRTM$_{MDT}$	21488	1.14	0.76	-1.06	9.99	1.40	0.96	-6.75	156.21	4.19	-12.16	154.23	0.27	2.05	0.04	3.94
SRTM$_{EGM96}$	21631	1.14	0.76	-1.06	9.99	2.65	2.20	-6.72	157.81	4.24	-10.48	155.83	1.52	2.36	1.30	4.22
ACE2$_{MDT}$	20385	1.14	0.76	-1.06	9.99	1.17	0.82	-6.85	186.02	3.96	-12.74	184.04	0.03	1.74	-0.08	3.76
ACE2$_{EGM96}$	20421	1.14	0.76	-1.06	9.99	2.45	2.10	-6.70	187.62	4.01	-11.08	185.65	1.31	2.05	1.19	4.00
MERITDEM$_{MDT}$	21556	1.13	0.76	-1.06	9.99	2.38	1.97	-5.50	141.54	3.31	-7.81	139.56	1.24	1.49	1.19	3.27
MERITDEM$_{EGM96}$	21557	1.13	0.76	-1.06	9.99	3.67	3.30	-4.17	143.15	3.36	-6.12	141.17	2.54	2.62	2.48	3.96
ASTGTM v003$_{MDT}$	21656	1.14	0.76	-0.45	9.99	8.59	7.40	-1.23	162.16	5.66	-7.29	160.19	7.46	7.54	6.37	9.51
ASTGTM v003$_{EGM96}$	21656	1.14	0.76	-0.45	9.99	9.89	8.72	0.00	163.77	5.65	-5.64	161.79	8.75	8.79	7.66	10.54
AW3D30$_{MDT}$	18262	1.18	0.77	-1.06	9.99	1.68	1.17	-6.27	116.48	3.40	-10.24	116.49	0.50	1.92	0.25	3.09
AW3D30$_{EGM96}$	18345	1.18	0.77	-1.06	9.99	2.94	2.43	-6.97	117.63	3.48	-8.56	117.65	1.77	2.37	1.52	3.55
TanDEM-X$_{MDT}$	20719	1.12	0.75	-1.06	9.99	2.28	0.75	-5.40	153.30	4.08	-8.86	151.32	1.15	1.42	0.44	3.89
TanDEM-X$_{EGM96}$	20724	1.12	0.75	-1.06	9.99	3.56	2.61	-3.79	154.90	4.11	-7.17	152.93	2.44	2.51	1.73	4.45
Copernicus DEM$_{MDT}$	18925	1.22	0.78	-0.45	9.99	2.31	1.20	-4.15	159.44	4.35	-8.90	157.46	1.09	1.49	0.25	4.15

Copernicus DEM$_{EGM2008}$	18925	1.22	0.78	-0.45	9.99	3.51	2.40	-2.92	161.01	4.35	-7.56	159.03	2.29	2.39	1.44	4.61
FABDEM$_{MDT}$	16736	1.24	0.78	-0.45	9.99	1.79	1.10	-0.97	99.53	3.47	-8.71	99.55	0.54	0.90	0.24	3.16
FABDEM$_{EGM2008}$	18771	1.22	0.78	-0.45	9.99	2.96	2.26	-0.92	150.50	3.68	-7.39	148.53	1.74	1.85	1.39	3.78
CoastalDEM v2.1$_{MDT}$	21540	1.13	0.76	-1.06	9.99	1.13	0.86	-4.08	126.31	3.27	-10.41	124.33	-0.00	1.03	-0.01	3.01
CoastalDEM v2.1$_{EGM96}$	21540	1.13	0.76	-1.06	9.99	2.42	2.10	-2.39	127.91	3.32	-8.71	125.94	1.29	1.56	1.29	3.28
GLL-DTM v2$_{MDT (this study)}$	19250	1.00	0.68	-0.45	9.99	0.97	0.79	-1.78	9.61	0.96	-9.39	6.10	-0.02	0.45	0.08	0.83
GLL-DTM v2$_{MDT (original)}$	19311	0.99	0.68	-0.45	9.99	1.14	0.95	-1.40	9.88	1.07	-8.90	6.55	0.14	0.53	0.25	0.84
DeltaDTM v1$_{MDT}$	15681	1.12	0.78	-0.45	9.99	0.93	0.72	-1.43	8.44	1.02	-8.94	4.99	-0.18	0.48	-0.07	0.86
DeltaDTM v1$_{EGM2008}$	18704	1.13	0.78	-0.45	9.99	2.15	1.90	-0.98	9.87	1.16	-7.58	7.65	1.02	1.18	1.10	1.35
DeltaDTM v1.1$_{MDT}$	18890	1.22	0.78	-0.45	9.99	0.94	0.58	-3.55	28.43	1.62	-8.90	26.45	-0.28	0.54	-0.20	1.04
DeltaDTM v1.1$_{EGM2008}$	18890	1.22	0.78	-0.45	9.99	2.14	1.75	-2.32	30.00	1.65	-7.67	28.02	0.92	1.09	1.00	1.36

Supplementary Table 5. Performance of local and global DEMs in the Vietnamese Mekong Delta and its adjacent provinces validated by local point elevations ≤10 m for versions referenced to mean dynamic topography (MDT) and their original vertical reference. N – number of spot heights in the study area, with no-data values excluded for each DEM, respectively; Mean spot – mean point elevation in the study area; Median spot – median point elevation in the study area; Min. spot – minimum point elevation in the study area; Max. spot – maximum point elevation in the study area; Mean DEM – mean DEM elevation in the study area; Median DEM – median DEM elevation in the study area; Min. DEM – minimum DEM elevation in the study area; Max. DEM – maximum DEM elevation in the study area; σ DEM – standard deviation of DEM elevation in the study area; HR – height residual; MAE – mean absolute error; RMSE – root mean square error.

Discussion

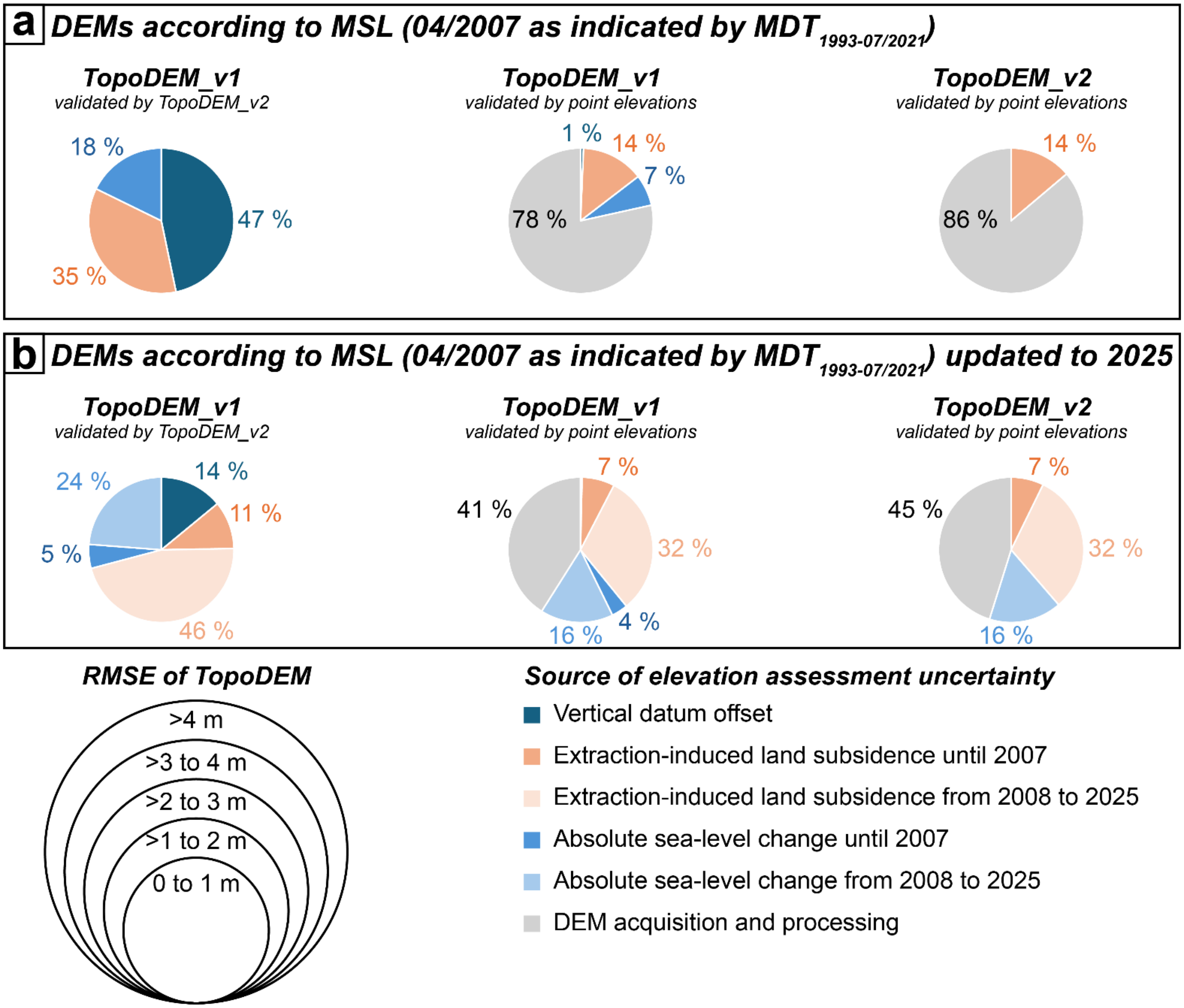

Supplementary Figure 4. Quantification and attribution of uncertainties associated with the local TopoDEM v1 and v2 in their performance to correctly quantify the elevation of the Vietnamese Mekong Delta. Quantifications were conducted relative to root mean square error (RMSE) of TopoDEM_v1 in comparison to TopoDEM_v2 based on its original vertical reference as well as in comparison to point elevations. Sources of elevation assessment uncertainty include vertical datum offset, inaccuracy resulting from DEM acquisition and processing, as well as time since DEM generation over which land subsidence and sea-level change have resulted in elevation change. (a) Relative uncertainties for DEMs referenced to mean sea level as indicated by mean dynamic topography (MDT), which provide the local sea level average over the period 1993–07/2021, corresponding to April 2007. (b) Relative uncertainties for DEMs referenced to mean sea level as indicated by mean dynamic topography (MDT), including land subsidence and sea-level rise since April 2007. For these tentative calculations, simulated, non-linear, extraction-induced land subsidence[3,4], tide-gauge observations[5] and IPCC-projections of sea-level rise[6–8] were used.

Year	Extraction-induced land subsidence (cm/yr)	Sea-level change (cm/yr)
2000	0.56	0.36
2001	0.58	0.36
2002	0.62	0.36
2003	0.68	0.36
2004	0.70	0.36
2005	0.73	0.36
2006	0.74	0.36
2007	0.76	0.36
Total (2000–2007)	*5.36*	*2.88*
2008	0.78	0.36
2009	0.79	0.36
2010	0.84	0.36
2011	0.84	0.45
2012	0.85	0.45
2013	0.86	0.45
2014	0.86	0.45
2015	0.87	0.45
2016	0.88	0.45
2017	0.88	0.45
2018	0.89	0.45
2019	0.89	0.45
2020	0.89	0.45
2021	0.89	0.49
2022	0.89	0.49
2023	0.89	0.49
2024	0.89	0.49
2025	0.89	0.49
Total (2008–2025)	*15.59*	*8.03*

Supplementary Table 6. Simulated, non-linear, extraction-induced land subsidence[3,4] (B1 scenario of Minderhoud et al.[4]), and annual rates of local, delta-average sea-level rise estimated from PSMSL data of Vung Tau tide-gauge observations (1979–2010)[5] and IPCC AR6 total rates of sea-level change[6–8] used to obtain tentative estimates of non-linear impact of elevation change due to vertical land motion (e.g. extraction-induced land subsidence) and sea-level change affecting DEM actuality.

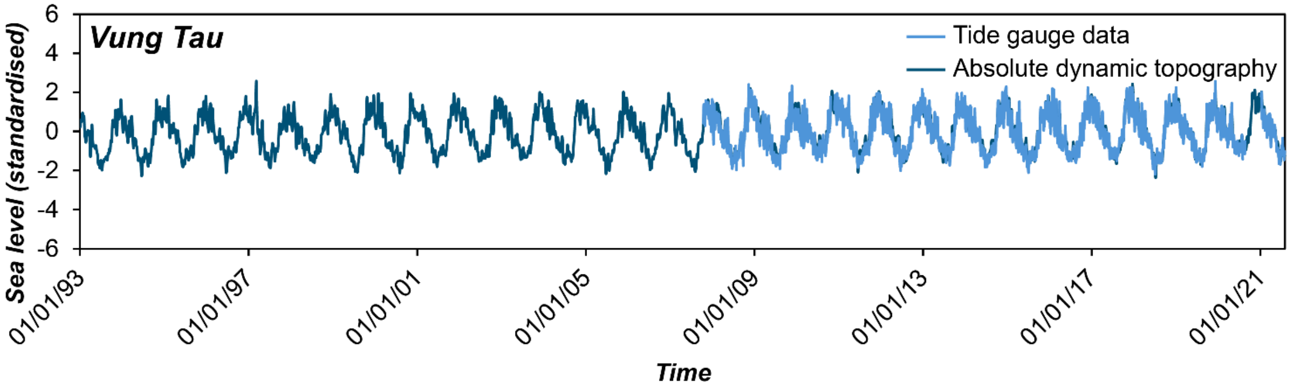

Supplementary Figure 5. Comparison between altimetry-based and tide-gauge based sea-level measurements for the location of Vung Tau tide gauge. Both records were standardised to ensure comparability. Altimetry data was obtained from ref. [9] and tide gauge observations from ref. [10].

Sea-level record	N	Mean	Median	Min.	Max.	Max. negative HR	Max. positive HR	Mean error	MAE	Median error	RMSE	Pearson coefficient	R^2
Tide gauge	4849	-0.05	-0.11	-2.19	2.56	-1.23	1.24	0.12	0.26	0.13	0.33	0.95	0.90
Absolute dynamic topography	4849	-0.03	-0.05	-2.37	2.58								

Supplementary Table 7. Statistics for standardised records of altimetry-based and tide-gauge based sea-level measurements for the location of Vung Tau tide gauge over the period of 16/10/2007–31/07/2021. The start date is limited by the availability of the tide gauge information while the end date indicates the time until which altimetry-based sea-level information is included into the MDT product[1] applied in this study. Altimetry data was obtained from ref. [9] and tide gauge observations from ref. [10].

References

1. Jousset, S., Mulet, S., Greiner, E., Wilkin, J., Vidar, L., Dibarboure, G. & Picot, N. New Global Mean Dynamic Topography CNES-CLS-22 Combining Drifters, Hydrological Profiles and High Frequency Radar Data. *ESS Open Archive*, DOI: https://doi.org/10.22541/essoar.170158328.85804859/v2 (2023).
2. Vernimmen, R. & Hooijer, A. New LiDAR-based elevation model shows greatest increase in global coastal exposure to flooding to be caused by early-stage sea-level rise. *Earth's Future* **11**(1), e2022EF002880, DOI: https://doi.org/10.1029/2022EF002880 (2023).
3. Minderhoud, P. S. J., Erkens, G., Pham, V. H., Bui, V. T., Erban, L., Kooi, H. & Stouthamer, E. Impacts of 25 years of groundwater extraction on subsidence in the Mekong delta, Vietnam. *Environ. Res. Lett.* **12**(6), 064006, DOI: https://doi.org/10.1088/1748-9326/aa7146 (2017).
4. Minderhoud, P. S. J., Middelkoop, H., Erkens, G. & Stouthamer, E. Groundwater extraction may drown mega-delta: projections of extraction-induced subsidence and elevation of the Mekong delta for the 21st century. *Environ. Res. Commun.* **2**(1), 011005, DOI: https://doi.org/10.1088/2515-7620/ab5e21 (2020).
5. Permanent Service for Mean Sea Level (PSMSL). Permanent Service for Mean Sea Level – Tide Gauge Data: Vung Tau, https://psmsl.org/data/obtaining/stations/1495.php (2025).
6. Fox-Kemper, B., Hewitt, H. T., Xiao, C., Aðalgeirsdóttir, G., Drijfhout, S. S., Edwards, T. L., Golledge, N. R., Hemer, M., Kopp, R. E., Krinner, G., Mix, A., Notz, D., Nowicki, S., Nurhati, I. S., Ruiz, L., Sallée, J.-B., Slangen, A. B. A. & Yu, Y. Ocean, Cryosphere and Sea Level Change. In: Climate Change 2021: The Physical Science Basis. Contribution of Working Group I to the Sixth Assessment Report of the Intergovernmental Panel on Climate Change, edited by Masson-Delmotte, V., Zhai, P., Pirani, A., Connors, S. L., Péan, C., Berger, S., Caud, N., Chen, Y., Goldfarb, L., Gomis, M. I., Huang, M., Leitzell, K., Lonnoy, E., Matthews, J. B. R., Maycock, T. K., Waterfield, T., Yelekçi, O., Yu, R., and Zhou, B., Cambridge University Press, Cambridge, 9-1–9-257 (2021).
7. Garner, G. G., Hermans, T., Kopp, R. E., Slangen, A. B. A., Edwards, T. L., Levermann, A., Nowikci, S., Palmer, M. D., Smith, C., Fox-Kemper, B., Hewitt, H. T., Xiao, C., Aðalgeirsdóttir, G., Drijfhout, S. S., Edwards, T. L., Golledge, N. R., Hemer, M., Kopp, R. E., Krinner, G., Mix, A., Notz, D., Nowicki, S., Nurhati, I. S., Ruiz, L., Sallée, J.-B., Yu, Y., Hua, L., Palmer, T. & Pearson, B. IPCC AR6 Sea-Level Rise Projections, Version 20210809, PO.DAAC, CA, USA, Dataset, https://podaac.jpl.nasa.gov/announcements/2021-08-09-Sea-level-projections-from-the-IPCC-6th-Assessment-Report (2021).
8. Garner, G. G., Kopp, R. E., Hermans, T., Slangen, A. B. A., Koubbe, G., Turilli, M., Jha, S., Edwards, T. L., Levermann, A., Nowikci, S., Palmer, M. D. & Smith, C. Framework for Assessing Changes To Sea-level (FACTS), Geoscientific Model Development, *Zenodo* https://zenodo.org/record/6419954 (2022).
9. E.U. Copernicus Marine Service Information (CMEMS). CLS. Global Ocean Gridded L 4 Sea Surface Heights And Derived Variables Reprocessed 1993 Ongoing, *Marine Data Store (MDS)* https://doi.org/10.48670/moi-00148 (2025).
10. University of Hawai'i Sea Level Center (UHSLC). 383 Vung Tau, Viet Nam: Quality controlled station data (research quality), UHSLC https://uhslc.soest.hawaii.edu/stations/?stn=383#levels (2025).
